# JASPAR 2026: expansion of transcription factor binding profiles and integration of deep learning models

**DOI:** 10.1093/nar/gkaf1209

**Published:** 2025-12-02

**Authors:** Damla Ovek Baydar, Ieva Rauluseviciute, Dina R Aronsen, Romain Blanc-Mathieu, Ine Bonthuis, Herman de Beukelaer, Katalin Ferenc, Alice Jegou, Vipin Kumar, Roza Berhanu Lemma, Jérémy Lucas, Mathis Pochon, Chang M Yun, Vivekanandan Ramalingam, Salil Sanjay Deshpande, Aman Patel, Georgi K Marinov, Austin T Wang, Alejandro Aguirre, Jaime A Castro-Mondragon, Damir Baranasic, Jeanne Chèneby, Sveinung Gundersen, Morten Johansen, Aziz Khan, Marieke L Kuijjer, Eivind Hovig, Boris Lenhard, Albin Sandelin, Klaas Vandepoele, Wyeth W Wasserman, François Parcy, Anshul Kundaje, Anthony Mathelier

**Affiliations:** Norwegian Centre for Molecular Biosciences and Medicine (NCMBM), Nordic EMBL Partnership, University of Oslo, Oslo 0318, Norway; Norwegian Centre for Molecular Biosciences and Medicine (NCMBM), Nordic EMBL Partnership, University of Oslo, Oslo 0318, Norway; Norwegian Centre for Molecular Biosciences and Medicine (NCMBM), Nordic EMBL Partnership, University of Oslo, Oslo 0318, Norway; Laboratoire Physiologie Cellulaire et Végétale, Univ. Grenoble Alpes, CNRS, CEA, INRAE, IRIG-DBSCI-LPCV, Grenoble F-38054 17 avenue des martyrs, France; Norwegian Centre for Molecular Biosciences and Medicine (NCMBM), Nordic EMBL Partnership, University of Oslo, Oslo 0318, Norway; Department of Plant Biotechnology and Bioinformatics, Ghent University, 9051, Ghent, Belgium; Center for Plant Systems Biology, VIB 9051 Ghent, Belgium; Norwegian Centre for Molecular Biosciences and Medicine (NCMBM), Nordic EMBL Partnership, University of Oslo, Oslo 0318, Norway; Laboratoire Physiologie Cellulaire et Végétale, Univ. Grenoble Alpes, CNRS, CEA, INRAE, IRIG-DBSCI-LPCV, Grenoble F-38054 17 avenue des martyrs, France; Norwegian Centre for Molecular Biosciences and Medicine (NCMBM), Nordic EMBL Partnership, University of Oslo, Oslo 0318, Norway; Norwegian Centre for Molecular Biosciences and Medicine (NCMBM), Nordic EMBL Partnership, University of Oslo, Oslo 0318, Norway; Laboratoire Physiologie Cellulaire et Végétale, Univ. Grenoble Alpes, CNRS, CEA, INRAE, IRIG-DBSCI-LPCV, Grenoble F-38054 17 avenue des martyrs, France; Laboratoire Physiologie Cellulaire et Végétale, Univ. Grenoble Alpes, CNRS, CEA, INRAE, IRIG-DBSCI-LPCV, Grenoble F-38054 17 avenue des martyrs, France; Department of Chemical Engineering, Stanford University, Stanford, CA 94305,United States; Department of Genetics, Stanford University, Stanford, CA 94305,United States; Institute for Computational and Mathematical Engineering (ICME), Stanford University, Stanford, CA 94305,United States; Department of Computer Science, Stanford University, Stanford, CA 94305,United States; Department of Genetics, Stanford University, Stanford, CA 94305,United States; Department of Computer Science, Stanford University, Stanford, CA 94305,United States; Department of Medical Genetics, University of British Columbia, Vancouver, BC V6T 1Z3,Canada; Centre for Molecular Medicine and Therapeutics, Department of Medical Genetics, BC Children’s Hospital Research Institute, University of British Columbia, BC V5Z 4H4 Vancouver 950 W 28th Ave, Canada; Norwegian Centre for Molecular Biosciences and Medicine (NCMBM), Nordic EMBL Partnership, University of Oslo, Oslo 0318, Norway; Akershus University Hospital, Department of Clinical Molecular Biology, Unit for Precision Medicine, Lørenskog, 1478, Norway; Division of Electronics, Ruđer Bošković Institute, 10000 Zagreb Bijenička cesta, Croatia; MRC Laboratory of Medical Sciences, London W12 0NN Du Cane Road, United Kingdom; Institute of Clinical Sciences, Faculty of Medicine, Imperial College London, Hammersmith Hospital Campus, London W12 0NN Du Cane Road, United Kingdom; Department of Biosciences, University of Oslo, Oslo 0316, Norway; Department of Biosciences, University of Oslo, Oslo 0316, Norway; Department of Biosciences, University of Oslo, Oslo 0316, Norway; Department of Computational Biology, Mohamed bin Zayed University of Artificial Intelligence (MBZUAI), Abu Dhabi, UAE; Norwegian Centre for Molecular Biosciences and Medicine (NCMBM), Nordic EMBL Partnership, University of Oslo, Oslo 0318, Norway; iCAN Flagship in Digital Precision Cancer Medicine, University of Helsinki, Helsinki, 00014,Finland; Department of Biochemistry and Developmental Biology, University of Helsinki, Helsinki, 00014,Finland; Department of Biosciences, University of Oslo, Oslo 0316, Norway; MRC Laboratory of Medical Sciences, London W12 0NN Du Cane Road, United Kingdom; Institute of Clinical Sciences, Faculty of Medicine, Imperial College London, Hammersmith Hospital Campus, London W12 0NN Du Cane Road, United Kingdom; Department of Biology and Biotech Research and Innovation Centre, University of Copenhagen, Ole Maaløes Vej 5, Copenhagen DK2200 N, Denmark; Department of Plant Biotechnology and Bioinformatics, Ghent University, 9051, Ghent, Belgium; Center for Plant Systems Biology, VIB 9051 Ghent, Belgium; Center for AI & Computational Biology, VIB, Ghent 9051, Belgium; Department of Medical Genetics, University of British Columbia, Vancouver, BC V6T 1Z3,Canada; Centre for Molecular Medicine and Therapeutics, Department of Medical Genetics, BC Children’s Hospital Research Institute, University of British Columbia, BC V5Z 4H4 Vancouver 950 W 28th Ave, Canada; Laboratoire Physiologie Cellulaire et Végétale, Univ. Grenoble Alpes, CNRS, CEA, INRAE, IRIG-DBSCI-LPCV, Grenoble F-38054 17 avenue des martyrs, France; Department of Genetics, Stanford University, Stanford, CA 94305,United States; Department of Computer Science, Stanford University, Stanford, CA 94305,United States; Norwegian Centre for Molecular Biosciences and Medicine (NCMBM), Nordic EMBL Partnership, University of Oslo, Oslo 0318, Norway; Department of Medical Genetics, Institute of Clinical Medicine, Oslo University Hospital and University of Oslo, Oslo 0318, Norway; Bioinformatics in Life Science (BiLS) initiative, Department of Pharmacy, University of Oslo, Oslo 0316, Norway

## Abstract

JASPAR (https://jaspar.elixir.no/) is an open-access database that has provided high-quality, manually curated, and non-redundant DNA binding profiles for transcription factors (TFs) as position frequency matrices (PFMs) for over 20 years. We expanded the CORE (306 new profiles, 12% increase) and UNVALIDATED (433, 60% increase) collections with new PFMs and updated 13 existing profiles. We updated the TF binding site predictions and genome tracks for eight species. TF binding profile clusters and familial TF binding sites were updated accordingly. We integrate the inMOTIFin software to easily simulate regulatory sequences using JASPAR PFMs. To enrich TFs’ annotations, we provide scientific literature-based human TF target information. Notably, this release features a deep learning (DL) collection, providing a paradigm shift in modeling and characterizing TF–DNA interactions with 1259 BPNet models trained on *Homo sapiens* ENCODE chromatin immunoprecipitation followed by sequencing (ChIP-seq) datasets from 240 TFs and interpreted to reveal predictive motif patterns for the models. The motifs associated with the same TF were clustered to provide a summary of the binding properties, resulting in 240 primary and 113 alternative motif patterns in the DL collection. The JASPAR 2026 collections lay a foundation for future endeavors in genomic research, serving the scientific community in uncovering the mechanisms of gene regulation.

## Graphical Abstract

**Graphical Abstract:**
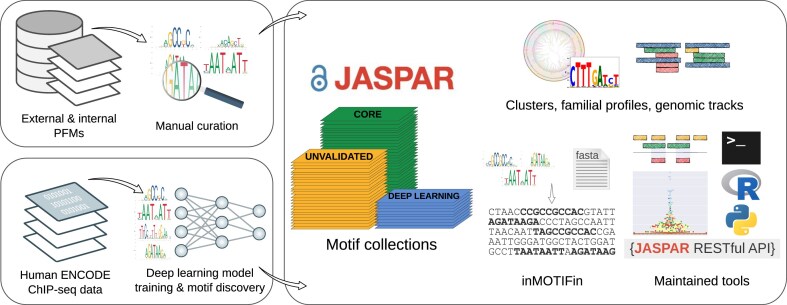

## Introduction

Transcription factors (TFs) are regulatory proteins that control gene transcription through *cis-*regulatory elements (CREs) such as promoters and enhancers. Although different types of TFs exist, this paper focuses on those that bind DNA in a sequence-specific manner [1]. For simplicity, we refer to them as TFs. The sequence-specific DNA binding of TFs is achieved through their DNA-binding domains (DBDs), which interact with the DNA at TF binding sites (TFBSs) [1]. While TFs recognize short DNA sequences, their binding activity in complex organisms is highly context-dependent [2]. In addition to the genomic sequence patterns recognized by TFs, their DNA occupancy is modulated by local chromatin accessibility, nucleosome positioning, DNA shape features, binding of other TFs, and the cooperation with other protein co-factors [3]. In many cases, TFs do not act in isolation but form cooperative complexes, stabilizing each other’s binding event [2, 4]. This combinatorial binding enables precise and dynamic regulation of gene expression across cell types and conditions.

Position weight matrices (PWMs), derived from position frequency matrices (PFMs), are the most common computational representations modeling how TFs interact with DNA. PWMs are quantitative summaries of a TF’s DNA-binding preferences, created by tallying the frequency, or log-likelihood score, of each nucleotide at every position within a set of experimentally observed TF–DNA interactions. These matrix models offer significant utility in various computational analyses, including the assessment of TFBS enrichment in regulatory regions [5], prediction of the impact of mutations in CREs, and guidance for *in vitro* mutagenesis experiments [6, 7]. Several open-access databases (e.g. CIS-BP [8], HOCOMOCO [9, 10], and JASPAR [11]) collect and store PFMs and PWMs.

JASPAR is an open-access database that provides manually curated, non-redundant TF binding profiles, primarily as PFMs across various taxonomic groups. Since its initial release in 2004 [12], JASPAR has become a standard resource in computational regulatory genomics due to its commitment to high-quality, accessible data and continuous expansion of content and tools, including a focus on open science and ease of use.

Despite their widespread use, PFMs have well-known limitations. They assume nucleotides at each position within TFBSs contribute independently to binding, even though interactions between adjacent or distant bases can be critical [13]. They also do not inherently consider genomic context (e.g. cooperativity, nucleosome positioning, co-factor binding) [3]. We and others have developed machine learning approaches, including Markov models [13, 14], variable-order Bayesian trees [15], support vector machines [16], and gradient boosting of decision trees [17], to improve TF–DNA interaction predictions by detecting more complex patterns.

Artificial intelligence, particularly the use of deep learning (DL) convolutional neural networks (CNNs), has led to new models that provide a shift in TF–DNA interaction modeling [18–22]. DL models are becoming an established method for decoding the *cis-*regulatory grammar of genomes [21, 22]. These models excel at autonomously discerning intricate regulatory patterns, facilitating context-specific and precise predictions [21, 22].

As such, DL models are trained in a supervised manner, and researchers aim to interpret the sequence patterns that have been captured. The interpretability of the models is performed using explainable artificial intelligence methods [20]. For instance, some methods, such as DeepLIFT [23], assign contribution scores to input nucleotides to pinpoint the ones with predictive power for the model. Then, tools like TF-MoDISco use contribution scores to derive predictive DNA motifs, thereby enabling the interpretability of the models [24]. Combining base-pair resolution predictive accuracy of experimental TF binding patterns (e.g. chromatin immunoprecipitation followed by sequencing, ChIP-seq) with motif-level interpretability, DL models, such as BPNet [25], have become transformative for studying condition-specific gene regulation by capturing complex features, such as the spacing and orientation of TFBSs and TF cooperativity.

To complement the classical PFM representation for modeling of TF–DNA binding, we now introduce a “Deep Learning” (DL) collection of BPNet-trained models on *Homo sapiens* TF ChIP-seq data from the ENCODE [26, 27], providing high-resolution, curated TF binding predictions and interpreted models visualized as logos. In this release, we have integrated 1259 BPNet models, identifying 353 primary and alternative motif patterns for 240 TFs in the DL collection.

Additionally, we have updated and expanded our CORE and UNVALIDATED collections. We have added 686 new profiles (265 in the CORE collection and 421 in the UNVALIDATED). Forty-one profiles from the UNVALIDATED collection of the previous release now have orthogonal support and were promoted to the CORE collection. Finally, we have updated 13 profiles with new matrices and also updated the metadata of 62 profiles.

Moreover, we have updated our tools and integrated new software into JASPAR. We introduce a simulation tool, inMOTIFin, that enables users to generate motifs and create or modify regulatory sequences by inserting motif instances into sequences with precise control over their frequencies, positions, and co-occurrences [28]. We have updated the pyJASPAR and Bioconductor packages. Finally, we introduce TF targets derived from a dedicated large language model that extracts TF-target relationships from the scientific literature.

## Results

### Expansion and update of the classical TF binding profiles

**Figure 1.**
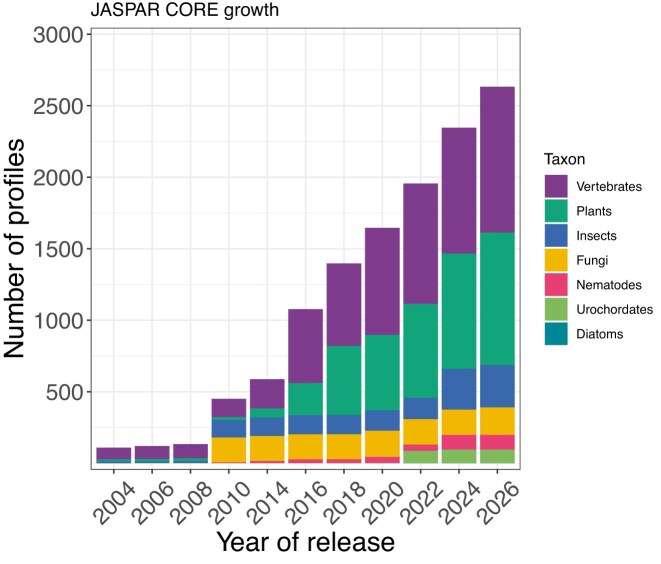
Overview of the growth of the number of profiles in the JASPAR CORE collection from the initiation of the database in 2004 to the latest 2026 release.

We performed manual curation of TF binding profiles using PFMs and position probability matrices from public resources (HOCOMOCO [10], ModERN [29], CIS-BP [30], Codebook/GRECO-BIT [31], and CAP-SELEX [32]). The collected set of profiles was complemented with PFMs that we generated through *de novo* motif enrichment analysis from ChIP-seq data from KRABopedia [33] and ModERN [29], as well as SMiLE-Seq, ChIP-seq, and GHT-SELEX from Codebook & GRECO-BIT [31] (see Supplementary Text for methodological details). Finally, we downloaded and processed CUT&RUN, ChIP-seq, DAP-seq, ampDAP-seq, and ChEC-seq data stored in GEO from individual studies (see Supplementary Table S1 for a complete list). JASPAR expert curators manually evaluated 11 565 profiles and selected the PFMs supported by orthogonal validation from the literature to add them or update former TF binding profiles in the JASPAR CORE collection. PFMs with high quality, but for which the curators did not find orthogonal support in the literature, were added to the JASPAR UNVALIDATED collection. In the current release, the JASPAR CORE collection has been complemented with 265 new binding profiles for four taxa: plants, 125 profiles (18% increase from the previous plants CORE collection), vertebrates, 121 profiles (16% increase), insects, four profiles (3.5% increase), and fungi, 15 profiles (8% increase) (Table 1 and Fig. 1). Furthermore, we promoted 41 profiles previously stored in the UNVALIDATED collection to the CORE collection after identifying orthogonal support from the literature (Table 1 and Fig. 1). Similarly, we complemented the UNVALIDATED collection with 871 new profiles for five taxa (Supplementary Table S2). Due to either profile redundancy or the underlying protein not being considered as a specific DNA-binding TF, we removed 11 profiles (seven from CORE and four from UNVALIDATED). After re-evaluation of the JASPAR profiles, we downgraded 12 profiles from CORE to UNVALIDATED due to insufficient literature support. Subsequently, we updated 13 profiles from the CORE collection with new, higher-quality PFMs. In addition to updating the matrices, we also updated existing profile metadata for 62 profiles in both collections.

**Table 1.** Summary of the JASPAR 2026 CORE collection update compared to the previous release

Taxonomic group	Non-redundant PFMs in JASPAR 2024	New non-redundant PFMs	Removed PFMs	Promoted PFMs (from UNVALIDATED to CORE)	Downgraded PFMs (from CORE to UNVALIDATED	Updated PFMs	Total non-redundant PFMs in JASPAR 2026
*Plants*	805	125	7	16	12	5	927
*Vertebrates*	879	121	−	19	−	7	1019
*Urochordata*	94	−	−	−	−	−	94
*Insects*	286	4	−	6	−	1	296
*Nematodes*	103	−	−	−	−	−	103
*Fungi*	178	15	−	−	−		193
*Diatoms*	1	−	−	−	−	−	1
CORE total	2346	265	7	41	12	13	2633

The current JASPAR 2026 release provides a total of 2633 and 1231 non-redundant TF DNA-binding profiles in the CORE and UNVALIDATED collections, respectively (Table 1, Fig. 1, Supplementary Table S2, and Supplementary Fig. S1).

### TF binding profile clusters, familial binding profiles, word clouds, and genomic tracks

JASPAR has provided PFM collections for over 20 years. Still, in addition to the collections, we include complementary features that enable users to interact with the data and gain insights into the characteristics of TF–DNA interactions and transcriptional regulation. We provide a clustering of TF binding profiles for each taxonomic group, and these groupings can be visualized and downloaded for the CORE collection and the combined CORE and UNVALIDATED collections. Specifically, users can inspect the similarity between TF binding profiles through radial and linear trees. As previously described, the TF binding profiles for TFs belonging to the same structural family or class are often very similar [1]. Therefore, we provide summaries of familial binding profiles obtained from a hierarchical clustering applied to the CORE collection in six main taxonomic groups. These familial binding profiles summarize similar TF binding profiles with a single PFM. In JASPAR 2026, we constructed 504 familial profiles using PFMs from the CORE collection (233 for vertebrates, 85 for insects, 69 for fungi, 55 for plants, 43 for nematodes, and 19 for urochordates). Users can access cluster and familial binding profile summaries at https://jaspar.elixir.no/matrix-clusters.

To supplement the information provided for each TF in JASPAR, we provide word clouds that summarize biological information explicitly associated with the TFs in the abstracts of scientific literature stored in PubMed. Since their introduction in 2022, we have updated the collection of word clouds and generated new ones for newly added profiles.

Lastly, we scanned the latest genome versions of *Arabidopsis thaliana, Caenorhabditis elegans, Ciona intestinalis, Danio rerio, Drosophila melanogaster, Homo sapiens, Mus musculus*, and *Saccharomyces cerevisiae* with TF binding profiles from the JASPAR 2026 CORE collection for the corresponding taxonomic group to predict potential TFBSs in the genomes. Using the predicted TFBSs and the familial binding profiles, we produced genomic tracks for familial binding sites by grouping TFBSs for TFs belonging to the same familial binding profile. Both TFBSs and familial binding site genomic tracks are available in multiple formats for users to visualize and interpret. Moreover, the TFBSs predicted in the mouse and human genomes with CORE vertebrate PFMs are available as native tracks in the UCSC Genome Browser [34].

### Large language model-based TF–target gene associations

TF–target gene (TF–TG) regulatory relationships are described in the literature. To facilitate the systematic identification of these interactions from published studies and complement the available TF-centric information provided in JASPAR, we designed a prompt and corresponding JSON output schema for ChatGPT-5 to extract TF–TG relationships from a collection of text-mined sentences in the ExTRI resource [35]. Manual curation of a set of 192 ExTRI-derived sentences specified the text referring to TFs and TGs, and a set of 350 relationships between the entities was generated automatically. Annotated TF–TG relationships were determined across four categories: positive regulation, negative regulation, neutral regulation (where no direction can be inferred from the text), and binding (where a TF binds to a DNA element associated with the target gene).

**Figure 2.**
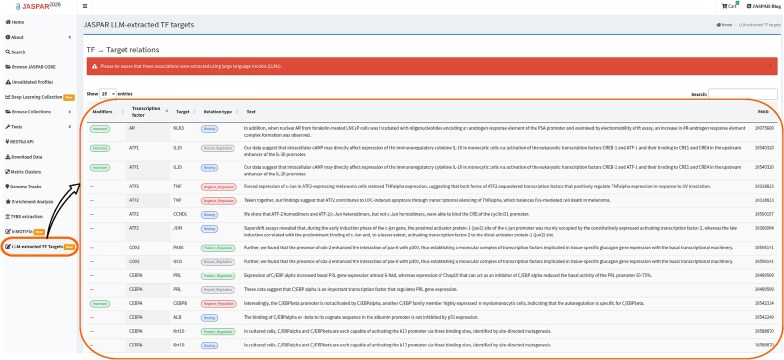
Web interface showing a table of annotated TF–TG relationships, including associated TF modifiers, source sentences, and PMIDs where the relationships were identified.

In addition, we captured TF modifiers, which provide contextual information about TF expression or activity in a given sentence. Five non-exclusive categories of modifiers were defined: mutant (the TF carries a mutation or is described as a non-wild-type form), increased expression or activity (e.g. overexpression *in vitro*), reduced expression or activity (e.g. down-regulation, inhibition, or knockdown), absent (e.g. knockout, complete depletion, loss of expression), and altered function (the TF is expressed in a non-canonical context such as an atypical tissue or cell type or acquires a novel DNA binding site). These modifiers provide essential context for interpreting TF–TG relationships (see Supplementary Text for details). Figure 2 illustrates how these annotated relationships are visualized within the JASPAR web interface.

The initial set of relationships was manually reviewed for accuracy (as were the modifiers described earlier), but the future expansions of the relationships produced will be fully automated. Therefore, we have indicated in the interface that these relationships are LLM-extracted; errors may occur. The accuracy of the LLM-generated annotations was 88% (308/350), with the most common mistakes being attributed to TF–TG relations that are influenced by the Increased, Absent, and Reduced TF modifiers, as well as relations that are described in hypothetical statements. Only the 308 correct TF–TG relationships were included in the online interface.

### Deep learning collection

**Figure 3.**
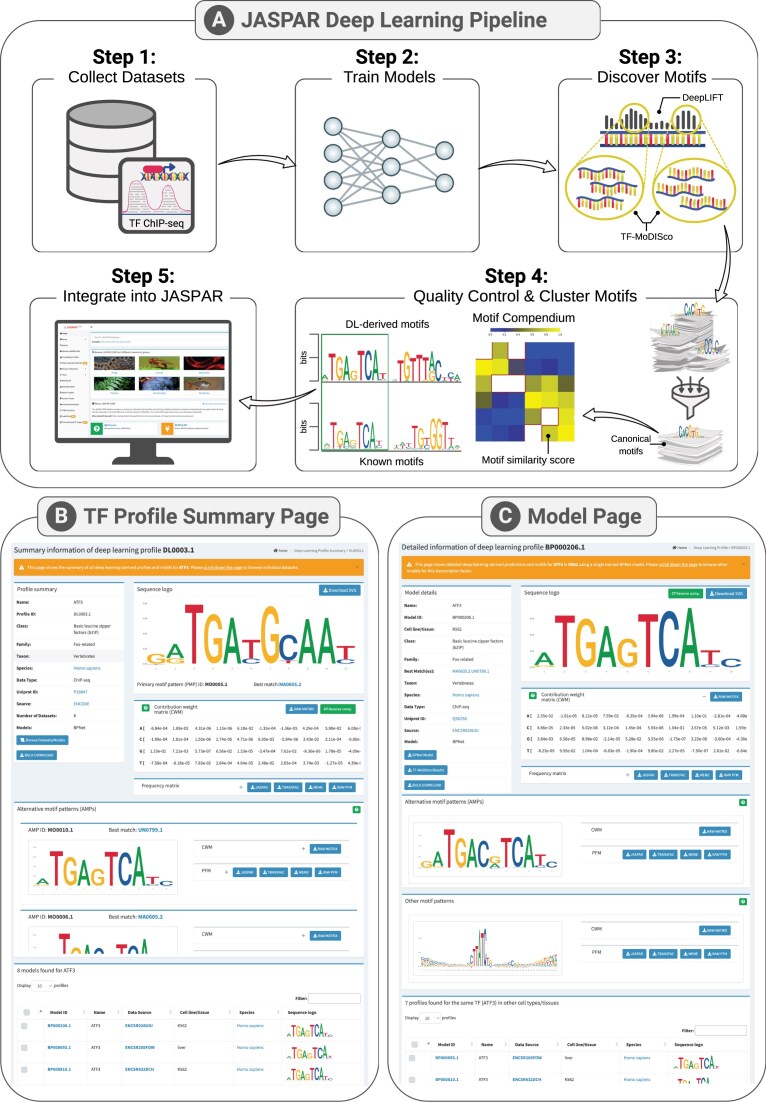
JASPAR 2026 introduces a deep learning collection. The top panel illustrates the comprehensive workflow. The bottom panels present screenshots of the TF summary profile page (left) and the model page (right).

**Figure 4.**
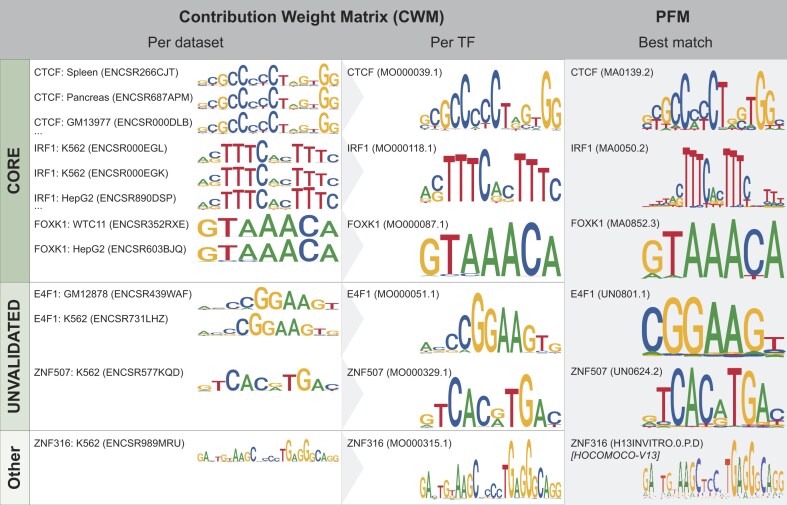
Examples of DL motif discovery, aggregation, and matching for CTCF, IRF1, FOXK1, E4F1, ZNF507, and ZNF316. (Left) Each CWM is derived from a BPNet model, each trained on an ENCODE TF ChIP-seq dataset, and revealed using TF-MoDISco. (Center) Individual CWMs are clustered and combined into aggregate CWMs per TF, using MotifCompendium. (Right) The closest matching PFM from JASPAR 2026, or other *in vitro*-derived PFM, was identified using MotifCompendium. (Top) CWMs that matched with a JASPAR 2026 CORE PFM. (Middle) CWMs that matched with a JASPAR 2026 UNVALIDATED PFM. (Bottom) CWMs that did not match with any JASPAR 2026 PFM, but matched with other *in vitro*-derived PFMs.

We retrieved *Homo sapiens* TF ChIP-seq datasets from ENCODE [26]. We trained a specific BPNet [25] model for each ChIP-seq dataset to predict the ChIP-seq genomic tracks at base-pair resolution from input DNA sequences (see Supplementary Fig. S2 for model performance metrics). We revealed the motifs most contributing to the accuracy of the models using DeepLIFT [23] and TF-MoDISco [24] (Fig. 3 and Supplementary Text). Next, we used the MotifCompendium tool (https://github.com/kundajelab/MotifCompendium) [36] to cluster all the discovered TF-MoDISco motifs per TF, thereby constructing TF binding profiles that summarize the most critical canonical binding pattern(s) across datasets for each TF. We applied several quality-control metrics to ensure that the identified motif patterns corresponding to the cognate TF binding profiles are supported by *in vitro* orthogonal evidence (Fig. 3 and Supplementary Text). These processing steps culminated in motifs for 240 TFs. As multiple motif patterns can be identified for the same TF, we provide them in dedicated summary profile pages (one per TF); each summary profile page is assigned an identifier DLXXXXXX.Y, where XXXXXX is the summary ID and Y is the version number (Fig. 3B). For each TF, we provide the primary motif pattern (PMP) and the alternative motif patterns (AMPs) (see Supplementary Text), culminating in 353 motif patterns with their best JASPAR 2026 PFM match (Fig. 4) for the 240 TFs (from 1 to 7 motif patterns per TF, Supplementary Fig. S3). All PMPs and AMPs are labeled as MOXXXXXX.Y, where XXXXXX is the motif pattern ID and Y is the version number, as above. The summary profiles are linked to 1259 BPNet models, each specific to an individual ChIP-seq dataset (Supplementary Table S3). The specific BPNet models are labeled BPXXXXXX.Y, where XXXXXX is the model ID and Y is the version number, as above (Fig. 3C). Similarly to the summary profiles, we provide all motif patterns revealed by TF-MoDISco and clustered by MotifCompendium for each dataset. All trained BPnet models are available to users, and we provide the TF binding profiles as PFMs and contribution weight matrices (CWMs), which are similar to PFMs but capture contribution scores to the model prediction aggregated across sequences.

We provide all the profiles and models as part of the JASPAR Deep Learning (DL) collection, which can be accessed through the left sidebar of the JASPAR website. This opens an interactive interface that displays a searchable list of TF binding profiles, along with advanced filtering options. Users can switch to a different view to explore the list of deep learning models. We provide a functionality to select models to scan their motifs against input sequences using the *tangermeme* framework [37]. Each TF summary profile has a dedicated page presenting metadata, a visualization of the PMPs and AMPs, the corresponding CWMs and PFMs, and a list of all models trained to predict the summary profile (Fig. 3B). Complementarily, we provide each BPnet model on a dedicated page, which includes metadata, the PMP, AMPs, and other motifs found by TF-MoDISco with logo visualization and matrix representations, and links to download the model and TF-MoDISco results for further analysis (Fig. 3C).

### JASPAR-associated tools

#### Incorporation of the regulatory sequence and motif simulation tool, inMOTIFin

When developing or evaluating computational methods to investigate the *cis-*regulatory grammar of genomes, it is critical to consider simulated data where the ground truth is known. To support such tasks, we developed inMOTIFin, a lightweight PFM and regulatory sequence simulation package [28]. In a nutshell, inMOTIFin can create PFMs with user-defined characteristics and generate DNA sequences with specific regulatory rules. To generate regulatory sequences, inMOTIFin inserts TFBSs in user-provided or random DNA sequences under flexible rules such as their positions, co-occurrences, and spacing. These features enable the design of synthetic data for benchmarking, analysis of TF binding cooperativity, and interpretation of computational models. We made it available on the JASPAR website to allow direct insertion of selected TF binding profiles. The user can set the number and length of random sequences to be generated or provide background sequences by uploading a FASTA file. When used from the website, each selected TF binding profile is inserted into the center of a randomly selected background by default. The user can also set the total number of motif instances per sequence. The output can be downloaded as a FASTA file, where the sequence headers include the background sequence and motif IDs, and a supporting BED file detailing the instances and positions of TFBS insertions. The package can also be installed locally and operated either through a command line or a Python interface (https://inmotifin.readthedocs.io/en/latest/, https://bitbucket.org/CBGR/inmotifin/src/main/). The stand-alone package has various additional features for tighter control over sequence simulations and modification. For seamless integration with JASPAR, the package supports the direct import of JASPAR profiles via the pyJASPAR module, given a user-provided set of matrix IDs.

#### pyJASPAR and R/bioconductor packages for JASPAR data access

The 2026 release of the JASPAR database can be accessed through its web interface athttps://jaspar.elixir.no and its RESTful API (https://jaspar.elixir.no/api/) [38].In addition, we maintain and update the pyJASPAR Python package (https://github.com/asntech/pyjaspar; https://doi.org/10.5281/zenodo.4485856) [39] and the JASPAR R/Bioconductor data package, which includes the new JASPAR2026 release (https://github.com/da-bar/JASPAR). They have both been updated to fetch the most up-to-date JASPAR data. The R/Bioconductor package now stores the latest and the two previous JASPAR releases. All JASPAR versions included in the package are now accessible through the AnnotationHub [40]. Additionally, pyJASPAR has been updated to enable the retrieval of models from the Deep Learning collection. This allows seamless integration of JASPAR data when working within Python or R.

## Conclusions and perspectives

As we present the 11th update of the JASPAR database, we expanded the JASPAR CORE collection by 12% (306 added or upgraded profiles; Table 1, Fig. 1). We have manually curated 11 565 profiles, derived from various databases and publications (Supplementary Table S1). It is becoming increasingly challenging to get big data sources to prepare motifs for manual curation, as we have nearly exhausted primary resources, such as GTRD or CIS-BP. The Codebook & GRECO-BIT consortium’s [31] most recent effort focused on less studied TFs and their interactions with the DNA. This significant effort provided an important data source for this JASPAR release with 2425 motifs derived from ChIP-seq, PBM, SMiLE-seq, HT-SELEX, and GHT-SELEX, which we considered for manual curation. Many TFs were assayed using all or multiple of these techniques. Therefore, we were able to use this resource to provide orthogonal support for many motifs across technologies. Efforts, such as Codebook & GRECO-BIT, are essential for the community and have enabled us to enrich our JASPAR collections, especially with the motifs of less studied TFs. This puts us a step forward toward having a complete list of curated profiles for human TFs.

We continued our efforts to add high-quality profiles, even if we were unable to find orthogonal support. The UNVALIDATED collection expanded by 60% (433 profiles added; Supplementary Fig. S1 and Supplementary Table S2). Notably, the majority of these profiles were CAP-SELEX-derived PFMs for TFs binding DNA as dimers [32]. We were able to find orthogonal confirmation for only a few of these dimer motifs, indicating the lack of literature and data investigating cooperativity between TFs with dedicated assays. We hope that, in the future, more such studies will be published to delve deeper into the more complex regulatory mechanisms that better capture how TFs cooperate to regulate transcription. Deep learning-based models will provide a paradigm shift in modeling TF cooperativity [25].

When preparing a new release of the JASPAR PFM collections, we aim to refine the already existing profiles. For the current release, we systematically revised 638 profiles in the UNVALIDATED collection and promoted 41 of them to the CORE collection. Finding orthogonal support in the literature remains a manual effort that can be time-consuming. The emerging development of large language models (LLMs) will likely ease and automate this process for curators in the near future. With JASPAR 2026, we introduced LLM-extracted TF targets, exemplifying how such tools can parse the scientific literature to extract deeper insights into complex transcriptional regulation by TFs.

With this update, we are pleased to fulfill a long-awaited promise and launch the JASPAR Deep Learning (DL) Collection. This release features 353 primary and alternative motif patterns summarizing the binding properties captured by deep learning models for 240 TFs. Importantly, JASPAR also provides the underlying 1259 BPNet models that were trained on specific ChIP-seq datasets. As such, the JASPAR DL collection provides state-of-the-art models predicting TF binding ChIP-seq signals at base pair resolution from DNA sequences. It complements the PFMs by providing unprecedented means to decode the *cis-*regulatory code of genomes. Nevertheless, we recognize that the binding profiles extracted from the trained models were validated by matching them with existing PFMs, thereby strengthening the importance of establishing high-quality resources that store TF binding profiles as PFMs. Indeed, high-quality, manually curated resources play a key role in training large models and should be maintained alongside more expressive representations [41, 42].

It is expected that interpreting deep learning models will leverage important characteristics such as the surrounding genomic context, TF cooperativity, and nucleosome positioning, which cannot be achieved by traditional motif discovery approaches [43–45]. Moreover, DL models can infer the impact of sequence variations on binding affinities through *in silico* mutagenesis. This approach has the potential to further our understanding of the molecular mechanisms driving diseases. With emerging tools like *tangermeme* and *ledidi* [37, 46], the community now has access to an ecosystem enabling the large-scale use of DL models to perform a multitude of tasks, such as interpreting the models, performing *in silico* mutagenesis, predicting TFBSs through hit calling, and generating regulatory sequences.

Integrating deep learning models into JASPAR opens new opportunities for future research endeavors. In this release, we have begun integrating models trained on *Homo sapiens* ChIP-seq datasets, providing a robust foundation for understanding human TF binding. We plan to expand the DL collection in the future by including other organisms and taxa, enhancing the depth and diversity of this collection.

Concurrently, the scientific community has increasingly focused on making deep learning models more accessible by collaborating worldwide on open-source projects. This update to JASPAR supports this community-driven initiative by integrating BPNet models directly into the database and providing seamless and direct access to them.

From its initial release, JASPAR has consistently provided the community with a high-quality, user-friendly resource that promotes open science. In summarizing this latest database update, we emphasize our unwavering commitment to evolving in tandem with technological advancements and scientific discoveries in the field. While expanding our data coverage and embracing new methodologies, we ensure the high quality, non-redundancy, and ease of use of the JASPAR database. This update reinforces our ongoing commitment to providing a resource at the forefront of transcription factor binding research.

## Supplementary Material

gkaf1209_Supplemental_Files

## Data Availability

JASPAR is an open-access database available at https://jaspar.elixir.no/.
