# Supplementary material for: JASPAR 2026: expansion of transcription factor binding profiles and integration of deep learning models
Source: Nucleic Acids Res. 2025 Dec 2;54(D1):D184–93. doi: 10.1093/nar/gkaf1209 (PMC12807658; doi:10.1093/nar/gkaf1209)
Supplement: gkaf1209_Supplemental_Files [file gkaf1209_supplemental_files.zip › JASPAR_2026_supplementary.pdf]

JASPAR 2026: expansion of transcription factor binding profiles and integration of deep learning models

Damla Övek Baydar[1,*], Ieva Rauluseviciute[1,*], Dina Ruud Aronsen[1,+], Romain Blanc-Mathieu[2,+], Ine Bonthuis[1,+], Herman de Beukelaer[3,4,+], Katalin Ferenc[1,+], Alice Jegou[2,+], Vipin Kumar[1,+], Roza Berhanu Lemma[1,+], Jérémy Lucas[2,+], Mathis Pochon[2,+], Chang M. Yun[5,+], Vivekanandan Ramalingam[6,+], Salil Sanjay Deshpande[7,+], Aman Patel[8], Georgi K. Marinov[6], Austin T. Wang[8], Alejandro Aguirre[9,10], Jaime A. Castro-Mondragon[1,11], Damir Baranasic[12,13,14], Jeanne Chèneby[15], Sveinung Gundersen[15], Morten Johansen[15], Aziz Khan[16], Marieke L. Kuijjer[1,17,18], Eivind Hovig[15], Boris Lenhard[13,14,§], Albin Sandelin[19,§], Klaas Vandepoele[3,4,20], Wyeth W. Wasserman[9,10,§], François Parcy[2,§], Anshul Kundaje[6,8,§], Anthony Mathelier[1,21,22,§]

[1] Norwegian Centre for Molecular Biosciences and Medicine (NCMBM), Nordic EMBL Partnership, University of Oslo, 0318 Oslo, Norway
[2] Laboratoire Physiologie Cellulaire et Végétale, Univ. Grenoble Alpes, CNRS, CEA, INRAE, IRIG-DBSCI-LPCV, 17 avenue des martyrs, F-38054, Grenoble, France
[3] Department of Plant Biotechnology and Bioinformatics, Ghent University, 9051 Ghent, Belgium
[4] Center for Plant Systems Biology, VIB, 9051 Ghent, Belgium
[5] Department of Chemical Engineering, Stanford University, CA, USA
[6] Department of Genetics, Stanford University, CA, USA
[7] Institute for Computational and Mathematical Engineering (ICME), Stanford University, CA, USA
[8] Department of Computer Science, Stanford University, CA, USA
[9] Department of Medical Genetics, University of British Columbia, Vancouver, BC, Canada
[10] Centre for Molecular Medicine and Therapeutics, Department of Medical Genetics, BC Children's Hospital Research Institute, University of British Columbia, 950 W 28th Ave, Vancouver, BC V5Z 4H4, Canada
[11] Akershus University Hospital, Department of Clinical Molecular Biology, Unit for Precision Medicine, Lørenskog, Norway
[12] Division of Electronics, Ruđer Bošković Institute, Bijenička cesta, 10000 Zagreb, Croatia
[13] MRC Laboratory of Medical Sciences, Du Cane Road, London, W12 0NN, UK
[14] Institute of Clinical Sciences, Faculty of Medicine, Imperial College London, Hammersmith Hospital Campus, Du Cane Road, London, W12 0NN, UK
[15] Department of Biosciences, University of Oslo, Oslo, Norway
[16] Department of Computational Biology, Mohamed bin Zayed University of Artificial Intelligence (MBZUAI), Abu Dhabi, UAE
[17] iCAN Flagship in Digital Precision Cancer Medicine, University of Helsinki, Helsinki, Finland
[18] Department of Biochemistry and Developmental Biology, University of Helsinki, Helsinki, Finland
[19] Department of Biology and Biotech Research and Innovation Centre, University of Copenhagen, Ole Maaløes Vej 5, DK2200 Copenhagen N, Denmark
[20] Center for AI & Computational Biology, VIB, 9051 Ghent, Belgium
[21] Department of Medical Genetics, Institute of Clinical Medicine, Oslo University Hospital and University of Oslo, Oslo, Norway
[22] Bioinformatics in Life Science (BiLS) initiative, Department of Pharmacy, University of Oslo, Oslo, Norway
[*] These authors contributed equally to this work as co-first authors
[+] These authors contributed equally to this work as co-second authors
[§] To whom correspondence should be addressed: anthony.mathelier@ncmbm.uio.no; francois.parcy@cea.fr; wyeth@cmmt.ubc.ca; akundaje@stanford.edu; albin@binf.ku.dk; b.lenhard@imperial.ac.uk

SUPPLEMENTARY TEXT

PFM data processing for manual curation

For manual curation, we retrieved PFMs from public resources and processed experimental datasets listed in Supplementary Table S1 (1–39). Specifically, we generated PFMs from experimental data using *de novo* motif discovery as described previously (Supplementary Text of JASPAR 2022 manuscript (JASPAR 2022: the 9th release of the open-access database of transcription factor binding profiles) (40). Our analysis pipeline summarizes both *de novo* discovered and downloaded PFMs to ease manual curation. The corresponding pipeline used for PFM data processing and preparation for manual curation is available at https://bitbucket.org/CBGR/jaspar_curation_pipeline/src/JASPAR2026/.

Codebook data download and preprocessing

The Codebook & GRECO-BIT data (5) were downloaded from the consortium's Zenodo repository at https://zenodo.org/records/10182957 (41). We used position probability matrices (PPMs) for downstream processing and curation. We discarded the 'not approved' motifs and considered the top 3 motifs per TF per data type (ChIP-seq, SMiLE-seq, HT-SELEX, GHT-SELEX, and PBM). In addition, we downloaded ChIP-seq, SMiLE-seq, and GHT-SELEX peak data associated with the Codebook & GRECO-BIT consortium (5) from GEO using the respective GEO accession IDs. We filtered out profiles that were derived from datasets not associated with sequence-specific DNA-binding TFs (see https://bitbucket.org/CBGR/jaspar_curation_pipeline/src/main/data/2026/nonTFs/). For more details on downloading the Codebook and GRECO-BIT data, as well as preprocessing instructions, please see https://bitbucket.org/CBGR/jaspar_curation_pipeline/src/main/data/2026/data_collection/GRECO-BIT_Codebook/.

HOCOMOCO data download and preprocessing

The Hocomoco v12 core collection data (8) was downloaded from https://hocomoco12.autosome.org/ in a single JASPAR-formatted file. We then split the data per TF and discarded the profiles that had already been curated for previous JASPAR releases. More details on downloading Hocomoco v12 data and preprocessing can be found at https://bitbucket.org/CBGR/jaspar_curation_pipeline/src/main/data/2026/data_collection/hocomoco/.

KRABopedia data download and preprocessing

The KRABopedia data containing peaks for Krüppel-associated box domain zinc finger proteins (KRAB-ZFPs) was downloaded from https://tronoapps.epfl.ch/web/krabopedia/download.php (9). KZFPs that were already curated for the JASPAR 2022 release were excluded from the curation. More details can be found at https://bitbucket.org/CBGR/jaspar_curation_pipeline/src/main/data/2026/data_collection/krabopedia/.

CISBP processing

MySQL tables containing DNA binding profiles from the CIS-BP Database Database Build 2.00 were downloaded from https://cisbp.ccbr.utoronto.ca/bulk.php (21). DNA binding profiles were filtered to exclude species already curated for previous releases of JASPAR, and only new species with direct experimental evidence were considered.

Individual dataset download from GEO

TF DNA binding data from individual studies (Supplementary Table S1) were downloaded from GEO (42) using their respective GEO accession IDs. The corresponding pipeline can be found at https://bitbucket.org/CBGR/jaspar_curation_pipeline/src/main/data/2026/data_collection/GEO_downloads/.

Manual curation

Plant profile validation by inference

We used PLAZA's (43) integrative method to infer orthologous TFs in *Arabidopsis thaliana*, *Oryza sativa ssp. japonica* and *Zea mays B73*. For each TF, the profiles to be validated were compared to known profiles (retrieved from JASPAR 2022) of orthologous TFs, based on the correlation scores obtained when clustering the matrices with RSAT (see below). As an initial filter, only those matches with Ncor1 > 0.5 were retained. We next manually evaluated this candidate list to remove false positives by visually comparing the sequence logos and retaining profiles that showed high similarity with their orthologous profile(s).

TF class and family classification inference

We obtained TF classification for vertebrate TFs from TFclass (44, 45). We used Plant-TFclass for plant TF classification (46). For TFs other than those in vertebrate and plant taxa, we inferred the classification hierarchy from their respective human homologs. Briefly, human ortholog information is obtained as follows: for Insects, nematodes, zebrafish, and Fungi, we utilized hyperlinks accessed from FlyBase (https://flybase.org/) (47), WormBase (https://wormbase.org/#012-34-5) (48), ZFIN (https://zfin.org/) (49), and PomBase/SGD (https://www.pombase.org/ or https://www.yeastgenome.org/) (50, 51) databases, respectively, jumping into the corresponding homologous TFs from human and/or other vertebrates. We also used Marrvel (http://marrvel.org/) and/or OrthoDB (https://www.orthodb.org/) to obtain TF homology (52, 53). In cases where no obvious homologous information is available from these databases, we inferred homology by performing a BLAST search of the protein sequence either within the NCBI's non-redundant database or the UniProtKB databases for humans. We took the top TF hit as the corresponding homolog and inferred TF classification from this homolog.

Matrix clustering

We clustered PFMs in the CORE collection and PFMs from the CORE+UNVALIDATED collections in six main taxa (vertebrates, plants, insects, nematodes, fungi, and urochordates). To perform the clustering, we used an updated stand-alone version of the RSAT matrix-clustering tool (https://github.com/jaimicore/matrix-clustering_stand-alone) (54). We used "`-m “Ncor” -W 4 -n 0.5`" parameters. On the JASPAR website, two visualizations are available: radial and linear trees. Profiles in the radial trees were annotated with TF structural information. We also provide additional output from the RSAT matrix-clustering tool for download.

Complementary data analysis

All complementary data were computed as described in the Supplementary Text of the JASPAR 2022 manuscript (40). Sequence logos and centrality plots are available for download on the website. Genomic tracks, TFBSs, and familial binding profiles are available at https://mencius.uio.no/JASPAR/JASPAR_genome_browser_tracks/, https://mencius.uio.no/JASPAR/JASPAR_TFBSs/, and

https://mencius.uio.no/JASPAR/JASPAR_familial_binding_sites/, respectively. LOLA databases are available on Zenodo at https://zenodo.org/uploads/16919600 and https://mencius.uio.no/JASPAR/JASPAR_LOLA_databases/.

Large language model-based TF-target knowledge

To generate the input sentences for the engineered ChatGPT prompt, we selected a subset of 199 sentences from ExTRI (55). Using ChatGPT 5 (model gpt-5-2025-08-07) (56) with the web_preview tool and reasoning effort set to medium via the API, we first performed entity linking by mapping the annotated entities in these sentences to NCBI Gene IDs (see Prompt 1 and JSON Schema 1 below). To ensure the model focused on sentence semantics rather than relying on domain knowledge, all entities (TFs, genes, and monomeric proteins) were masked, preserving repeated mentions of the same entity. For example, the sentence *"HIF-1 is considered the primary trans-acting factor for the erythropoietin (EPO) and vascular endothelial growth factor (VEGF) genes" (57)* was transformed into *"HIF-1 is considered the primary trans-acting factor for the [GENE_1] ([GENE_1]) and [GENE_2] ([GENE_2]) genes."*

As described in the main text, we captured TF modifiers in addition to TF–target gene relationships. The importance of contextual information provided by modifiers is illustrated in the following sentence from (58): "Inhibition of Oct-2 synthesis reduces TGF alpha expression in astroglial cells and delays the initiation of puberty." Incorporating modifiers allows us to extract the triplet: Reduced ⟶ Oct-2 ⟶ Negative regulation ⟶ TGF alpha, revealing that in this context, the reduction of Oct-2 down-regulates TGF alpha.

A subset of sentences with manually annotated relationships was used to iteratively develop the prompt and JSON schema for the relation extraction task. This subset was selected to capture varying levels of complexity in terms of the number of entities, relationships, and TF modifiers. For each iteration, ChatGPT's output was compared with the corresponding manual annotations of TF–TG relationships and TF modifiers. The specific model used was gpt-5-2025-08-07 with reasoning effort set to high via the API. The prompt was refined until, within this development subset, the model consistently reproduced the human annotations (see Prompt 2 and JSON Schema 2). The final optimized prompt was then applied to the complete set of 199 sentences to extract TF–TG relationships for JASPAR.

Prompt 1: Used for entity linking to NCBI Gene IDs.
Developer message:
You are a molecular biology and genomics expert. You will receive a JSON input with the following structure:

```
{
"text": string,
"query": string
}
```

Your task is to return a JSON object containing a single key, "gene_ids", whose value is a list of NCBI Gene IDs corresponding to gene or protein entities mentioned in the query, based on the context in the provided text.

Rules:
- Return only valid JSON, following the specified schema. No explanations, comments, or extra content.
- Always use and search online official genomics resources (such as NCBI (59), UniProt (60), GeneCards (61)) and biomedical literature (PubMed, PMC) for completing the task.

Steps:
1. Determine if the query contains one or more gene/protein entities.
2. For each entity, perform the following steps according to the rules:
   2.1. Determine if the entity is a single gene/protein as opposed to a gene/protein family or protein complex. Skip the entity if it is not a single gene/protein.
   2.2. Determine the most likely species for the gene/protein from the context in the input text.
   2.3. Search for the NCBI Gene ID of the identified and disambiguated gene/protein entity.
   2.4. If no valid NCBI Gene ID exists for an entity, skip such entity.
3. Return the identified NCBI Gene ID(s) as a list according to the JSON schema. If no IDs were identified, return an empty list.

Example 1:

```
{"text": "The tumor suppressor p53 regulates apoptosis.", "query": "p53"}
```

Output: `{"gene_ids": [7157]}`

Example 2:

```
{"text": "Sonic Hedgehog-induced activation of the Gli1 promoter is mediated by
GLI3.", "query": "mediated"}
```

Output: `{"gene_ids": []}`

Example 3:

```
{"text": "The BRCA1/2 genes are very important in the oncology field."
"query": "BRCA1/2"}
```

Output: `{"gene_ids": [672, 675]}`

User message (placeholders are denoted by {{ }}):

```
{
  "text": {{text}},
  "query": {{query}}
}
```

JSON Schema 1: Used to indicate output formatting for entity linking to NCBI Gene IDs

```
{
  "name": "ncbi_gene_ids",
  "strict": true,
  "schema": {
    "type": "object",
    "properties": {
      "gene_ids": {
        "type": "array",
            "description": "List of NCBI Gene IDs corresponding to the query gene(s). Empty if none are found.",
        "items": {
          "type": "integer"
        }
      }
    },
    "required": [
      "gene_ids"
    ],
    "additionalProperties": false
  }
}
```

Prompt 2: Used for extracting TF-TG relationships.
Developer message:
You're a molecular biologist who specializes in identifying relations between transcription factors and other transcription factors or target genes.

You will receive as input a sentence with two types of masked entities, each mentioned wrapped in brackets []:
- Transcription factors: Masked as `TRANSCRIPTION_FACTOR_ID`. These are transcription factor entities.
- Genetic: Masked as `GENETIC_ID`. These are non-transcription factor genes.

In the masked entities, ID corresponds to a number that defines the identity of such an entity. In your output, use the names of the masked entities without the enclosing brackets [].

Your task is to return a JSON list of all relations present where a transcription factor is targeting another transcription factor or genetic entity in the sentence.

For generating the JSON, you will also identify transcription factor modifiers. These categories indicate alterations in the activity or expression of a transcription factor entity. This alteration is not caused by another transcription factor or a genetic entity. Modifiers should be present either as an empty list or a list of modifiers whenever the entity is a transcription factor (`[TRANSCRIPTION_FACTOR_ID]`). Modifiers should be null when the entity is a genetic entity (`[GENETIC_ID]`).

There are four types of relations:

- Positive regulation
- Negative regulation
- Neutral regulation (no direction is specified)
- Binding (the transcription factor physically binds to the gene or a regulatory element of the gene, such as a promoter or enhancer)

User message: Masked sentences. For example:
The binding of [TRANSCRIPTION_FACTOR_1] and [TRANSCRIPTION_FACTOR_2], transcription factors implicated in [GENETIC_1] expression, in the [GENETIC_1] promoter was not affected by the methylation status of neighboring CpG sites.

JSON Schema 2: Used to indicate output formatting for relation extraction.

```
{
  "name": "tf_relations",
  "strict": true,
  "schema": {
    "type": "object",
    "properties": {
      "relations": {
        "type": "array",
        "items": {
          "type": "object",
          "properties": {
            "head": {
              "type": "object",
              "properties": {
                "entity": {
                  "type": "string",
                  "description": "Name of the transcription factor entity. It is always encoded inside brackets as [TRANSCRIPTION_FACTOR_(ID)], where ID is a number identifying the entity. The brackets [] are for recognition, but only the enclosed string should be taken into account."
                },
                "modifiers": {
                  "type": [
                    "array",
                    "null"
                  ],
                  "description": "Modifiers, if any, influencing the transcription factor entity. Must be an empty array if the child entity is a genetic entity.",
                  "items": {
                    "type": [
                      "string",
                      "null"
                    ],
                    "enum": [
                      "Mutant",
                      "New_Function",
```

```
                      "Absent",
                      "Reduced",
                      "Increased",
                      null
                    ],
                    "additionalProperties": false
                  }
                }
              },
              "required": [
                "entity",
                "modifiers"
              ],
              "additionalProperties": false,
              "description": "The transcription factor entity that is the source
(head) of the relation."
            },
            "child": {
              "type": "object",
              "properties": {
                "entity": {
                  "type": "string",
                    "description": "Name of the child entity. If the child is a
transcription  factor,  entity  is  always  encoded  inside  brackets  as
[TRANSCRIPTION_FACTOR_(ID)], where ID is a number identifying the entity. If the
child is a genetic entity, the name does not follow the bracket convention."
                },
                "modifiers": {
                  "type": [
                    "array",
                    "null"
                  ],
                           "description": "Modifiers, if any, influencing the
transcription factor entity. Must be an empty array if the child entity is a
genetic entity.",
                  "items": {
                    "type": [
                      "string",
                      "null"
                    ],
                    "enum": [
                      "Mutant",
                      "New_Function",
                      "Absent",
                      "Reduced",
                      "Increased",
                      null
                    ],
                    "additionalProperties": false
                  }
```

```
                }
              },
              "required": [
                "entity",
                "modifiers"
              ],
              "additionalProperties": false,
               "description": "The child (target) entity of the relation. If the child is a transcription factor, include modifiers; if genetic, modifiers is an empty array."
            },
            "type": {
              "type": "string",
              "enum": [
                "Positive_Regulation",
                "Negative_Regulation",
                "Neutral_Regulation",
                "Binding"
              ],
               "description": "The label for the relation between head and child entities."
            }
          },
          "required": [
            "head",
            "child",
            "type"
          ],
          "additionalProperties": false
        },
           "description":  "List of Relation objects representing relationships between transcription factors and other entities"
      }
    },
    "required": [
      "relations"
    ],
    "additionalProperties": false
  }
}
```

Deep learning collection preparation

Data processing and model training

Uniformly processed *H. sapiens* TF ChIP-seq datasets were obtained from the ENCODE portal (https://www.encodeproject.org/). Base-resolution signal tracks were generated from the 5′ ends of mapped reads following the removal of PCR duplicates and low-quality reads.

We adapted the BPNet architecture and training procedure from Avsec *et al.* (62) (https://github.com/kundajelab/bpnet-refactor). The model accepts a one-hot-encoded 2,114 bp input sequence and predicts strand-specific ChIP-seq signal profiles and total log counts over a central 1,000 bp window. The model predicts the residual signal relative to the control input tracks. The architecture comprises nine dilated convolutional layers with residual connections. Profile predictions are generated via an additional convolutional layer applied to the final dilated output, integrating the control signal profiles. Total counts are predicted by applying global average pooling, followed by a dense layer incorporating control total counts. The model is trained using a joint loss function that combines a single negative log-likelihood loss for the profile logits across both the strand and mean squared error for total counts.

Training data consisted of IDR-thresholded peaks and GC-matched non-peak regions in a 3:1 ratio. Outlier peaks with total signal exceeding 1.2× the total signal of the 99th percentile peak were excluded. Data augmentation included random jittering (up to 128 bp) and reverse-complement transformations. Models were trained using chromosome-based 5-fold cross-validation with no overlap between training, validation, and test sets.

Contribution score and motif discovery

Using the trained models, we calculated the contribution of each base in the peak regions of the dataset to the relative log fold change in predicted read counts relative to respective shuffled GC content-matched sequences using DeepLIFT/DeepSHAP (63). We focused on the contribution score from the predicted read counts due to its straightforward interpretability: the sum of contribution scores of all bases and positions of a peak region is equal to the relative log fold change in predicted read count of the peak region compared to a GC-matched, dinucleotide-shuffled reference region. Using the mean count DeepLIFT/DeepSHAP contribution scores across the five model-folds, we performed motif discovery using TF-MoDISco, identifying contribution weight matrices (CWMs) of length 50 bp (initial core motif length 20 bp, add initial flank of 5 bp, add final flanks of 10 bp), and 110 bp (initial core motif length 30 bp, add initial flank 20 bp, add final flank 20 bp), within a region of width 400 bp from the peak summit, and a maximum seqlet count of 50,000.

Model quality

Using the discovered CWMs, we manually selected models for which a known motif of the ChIP target TF was identified by comparing with publicly available motifs from JASPAR 2024 CORE (20), HOCOMOCO V13 Core (8), CIS-BP V3 (21), zinc finger bacterial one-hybrid (B1H) system (64, 65). Except for the ZNF models, which generally result in lower model performance, all models were also filtered for having greater than a median 0.5 Pearson correlation coefficient for the counts head across the model folds. This resulted in a set of 1,644 BPNet models that passed initial manual curation. Furthermore, we utilized MotifCompendium (https://github.com/kundajelab/motifcompendium) (66), a GPU-accelerated motif similarity, clustering, and management package for CWMs, to further refine our model selection. The MotifCompendium package calculates a similarity score between 0 and 1 for any pair of CWMs by calculating the information content (IC)-scaled, cosine similarity between two motifs across all possible alignments and reverse complement orientations, accelerated by GPU kernels (see details in (66)). To ensure high-quality models that capture TF binding properties, we selected BPNet models

that identified a motif matching a known motif of the ChIP target TF JASPAR 2026 CORE or UNVALIDATED collections, with a similarity score greater than 0.88. This resulted in a final set of 1,259 BPNet models and 14,575 CWMs across 240 TFs that passed model quality checks.

Annotation and clustering

The CWMs satisfying our quality control were annotated and clustered using MotifCompendium. For annotation, we divided each CWM into its ChIP target TFs. To ensure that the CWMs correspond to *bona fide* canonical motifs bound by the ChIP'ed TFs, we sought orthogonal evidence using *in vitro* data. Specifically, we identified, for each TF, the CWMs with orthogonal evidence of binding by matching with JASPAR 2026 CORE and UNVALIDATED PFMs (this publication), or publicly available *in vitro* experiment-derived motifs from HOCOMOCO V13 *in vitro (8)*, CIS-BP V3 (21), Codebook (5), and CAP-SELEX (11), with a similarity score greater than 0.88. This resulted in 1,822 confirmed, CWM-based motifs with orthogonal evidence of binding *in vitro*.

To create a non-redundant set of unique, annotated motifs, we performed clustering on the confirmed motifs per TF using MotifCompendium. For each TF, we calculated the pairwise similarity between all confirmed motifs of the TF. Then, we performed two rounds of recursive community detection clustering, using a weighted Constant Potts Leiden algorithm on the similarity matrix thresholded at 0.88, and a Densely Connected Components algorithm on the similarity matrix thresholded at 0.92. Each cluster was then averaged into a single representative CWM by taking the mean of its constituent CWMs. This resulted in 353 unique, orthogonally confirmed cluster CWMs. To compare and match the cluster CWMs with their most similar JASPAR 2026 PFMs, we calculated the pairwise similarity between all cluster CWMs and all JASPAR 2026 CORE and UNVALIDATED motifs per TF using MotifCompendium. A pair was considered a match if the similarity score was greater than 0.7, and the pair with the highest similarity score was selected as its best match. If no match was found, TF paralogs were considered. If no match was still found, the cluster CWM remained without a JASPAR 2026 match. 268 cluster CWMs had a direct match with a JASPAR 2026 CORE PFM; 23 with a JASPAR 2026 UNVALIDATED PFM; 8 with a JASPAR 2026 CORE or UNVALIDATED PFM of a paralog; and 8 did not match any JASPAR 2026 CORE or UNVALIDATED PFM but matched with other *in vitro*-experiment derived PFMs (8).

Motif occurrences

To translate CWMs into PFMs, we identified in-peak genomic motif occurrences of each CWM using FiNeMo (https://github.com/austintwang/finemo_gpu), a GPU-accelerated motif hit caller for identifying occurrences of CWM motifs within deep learning contribution scores. For each set of TF-MoDisCo CWMs and their original contribution scores, we identified motif occurrences within a region of width 1,000 bp from the peak summits and a sensitivity lambda of 0.7. To create PFMs for CWM motifs, the sequences of the CWM motif's occurrences were summed up to create its corresponding sequence instance count PFM. To create PFMs for the cluster CWMs, the PFMs of the cluster's constituent motifs were further summed up to generate the sequence instance count cluster PFM.

For further details on the deep learning collection process, please refer to (66).

Updates in the JASPAR software components

3+1D

Beyond maintaining the computational backend of the bi-yearly JASPAR database update (previously described as 3D - **d**iscovery, **d**ownstream, **d**eployment), we added a new **D**eep Learning component. Here we described the most important changes we made.

Discovery

For this release, we updated and simplified the discovery pipeline. Within this effort, we translated it to Nextflow (67), utilizing already existing nf-core modules (68) and creating additional local modules (https://bitbucket.org/CBGR/jaspar_curation_pipeline/src/JASPAR2026/). We also updated to use CentriMo (69) for the analysis of motif centrality, which is the parameter we rely on for selecting the best motif candidates from the analyzed datasets.

Downstream

We have introduced a new feature that enables motifs to be downgraded from CORE to UNVALIDATED after a thorough review of their validation by expert curators. Furthermore, we added taxon information to the matrix history table. The analysis pipeline used for this release can be found at https://bitbucket.org/CBGR/jaspar_downstream/src/JASPAR2026/.

Deployment

We retired the motif permutation and randomization online tools, as they became redundant with the addition of inMOTIFin. The website is deployed through Jenkins (https://www.jenkins.io/), following an Ansible playbook developed by ELIXIR Norway (https://github.com/elixir-oslo/jaspar-playbook), based on the deployment pipeline (https://bitbucket.org/CBGR/jaspar_deployment/src/jaspar2026/). ELIXIR Norway hosts the JASPAR website on Norwegian Research and Education Cloud (NREC) resources and provides maintenance and support services.

Deep Learning

We introduced a new database storing the DL collection and created a Nextflow-based (67) downstream analysis pipeline to streamline their analysis. The pipeline can be found here: https://bitbucket.org/CBGR/jaspar_dl_downstream/src/jaspar2026/. Input to this pipeline is: 1) a metadata sheet listing all the DL models to be integrated into JASPAR along with their metadata information such as data source, cell line/tissue of the experiment, and target TF name, 2) two metadata sheets listing all the motifs clustered using MotifCompendium (as described above) and listing cluster motifs, and 3) four H5 files, in TF-MoDISco format, storing CWMs and PFMs of all the cluster and model motifs to be integrated into JASPAR.

For each TF, we provide a summary page that includes all quality-controlled motif patterns. The summary pages are assigned an identifier DLXXXXXX`.Y per TF`. For each TF, we classified each cluster CWM into one of three categories: (i) matching a 2026 JASPAR CORE motif, (ii) matching a 2026 JASPAR UNVALIDATED motif, or (iii) matching an *in vitro*-derived motif from another database

(see above). Within each category, we ranked motif patterns by the number of motif instances in ChIP-seq peaks using FiNeMo (see above). The primary motif pattern (PMP) for a TF was defined as the top-ranked motif in category (i); if no motif was present in category (i), the PMP was the top-ranked motif in category (ii); and if neither (i) nor (ii) contained a motif, the PMP was the top-ranked motif in category (iii). All the other motifs were defined as alternative motif patterns (AMPs). All PMPs and AMPs were assigned an identifier `MOXXXXX.Y`.

All underlying BPnet models for all ChIP-seq datasets are also available in the JASPAR DL collection for each TF and are assigned an identifier BPXXXXXX.Y. When multiple motifs identified by a BPnet model for a given TF ChIP-seq experiment map to the same primary motif pattern of the TF, we selected the motif with the highest number of contributing seqlets.

Finally, the pipeline trims the motifs to focus on the main contributing nucleotides, produces CWM logos, generates PFM matrices for each motif in four different formats, and provides CWM matrices in raw format. The trimming strategy follows the implementation in the MotifCompendium tool (https://github.com/kundajelab/MotifCompendium/blob/main/MotifCompendium/utils/motif.py). Specifically, for each position in the CWMs, we computed the sum of absolute contribution values across nucleotides. The total contribution across the motif is then calculated, and positions are retained if their contribution exceeds a threshold of $1/L$ of the total sum, where L is the motif length. The leftmost and rightmost positions exceeding this threshold define the trimmed motif boundaries, and only positions within these boundaries are kept.

All four components are updated consistently to ensure optimal cross-talking and ease of use. We adhere to best software development practices to the best of our ability. For example, we develop containers to ensure reproducibility (https://hub.docker.com/repositories/cbgr), we use version control, automated checks, and unit tests. The code is often developed simultaneously by multiple team members using the JIRA management system and code reviews.

References

1. Nordin,A., Zambanini,G., Enar Jonasson,M., Weiss,T., van de Grift,Y., Pagella,P. and Cantù,C. (2025) Construction of an atlas of transcription factor binding during mouse development identifies popular regulatory regions. *Development*, **152**.

2. Weigel,B., Tegethoff,J.F., Grieder,S.D., Lim,B., Nagarajan,B., Liu,Y.-C., Truberg,J., Papageorgiou,D., Adrian-Segarra,J.M., Schmidt,L.K., *et al.* (2023) MYT1L haploinsufficiency in human neurons and mice causes autism-associated phenotypes that can be reversed by genetic and pharmacologic intervention. *Mol. Psychiatry*, **28**, 2122–2135.

3. Fixsen,B.R., Han,C.Z., Zhou,Y., Spann,N.J., Saisan,P., Shen,Z., Balak,C., Sakai,M., Cobo,I., Holtman,I.R., *et al.* (2023) SALL1 enforces microglia-specific DNA binding and function of SMADs to establish microglia identity. *Nat. Immunol.*, **24**, 1188–1199.

4. Razavi,R., Fathi,A., Yellan,I., Brechalov,A., Laverty,K.U., Jolma,A., Hernandez-Corchado,A., Zheng,H., Yang,A.W.H., Albu,M., *et al.* (2024) Extensive binding of uncharacterized human transcription factors to genomic dark matter. *bioRxiv*, 10.1101/2024.11.11.622123.

5. Jolma,A., Laverty,K.U., Fathi,A., Yang,A.W.H., Yellan,I., Vorontsov,I.E., Inukai,S., Kribelbauer-Swietek,J.F., Gralak,A.J., Razavi,R., *et al.* (2024) Perspectives on Codebook: sequence specificity of uncharacterized

human transcription factors. *bioRxiv*, 10.1101/2024.11.11.622097.

6. Vorontsov,I.E., Kozin,I., Abramov,S., Boytsov,A., Jolma,A., Albu,M., Ambrosini,G., Faltejskova,K., Gralak,A.J., Gryzunov,N., *et al.* (2024) Cross-platform DNA motif discovery and benchmarking to explore binding specificities of poorly studied human transcription factors. *bioRxiv*, 10.1101/2024.11.11.619379.

7. Jolma,A., Hernandez-Corchado,A., Yang,A.W.H., Fathi,A., Laverty,K.U., Brechalov,A., Razavi,R., Albu,M., Zheng,H., Codebook Consortium, *et al.* (2024) GHT-SELEX demonstrates unexpectedly high intrinsic sequence specificity and complex DNA binding of many human transcription factors. *bioRxivorg*, 10.1101/2024.11.11.618478.

8. Vorontsov,I.E., Eliseeva,I.A., Zinkevich,A., Nikonov,M., Abramov,S., Boytsov,A., Kamenets,V., Kasianova,A., Kolmykov,S., Yevshin,I.S., *et al.* (2024) HOCOMOCO in 2024: a rebuild of the curated collection of binding models for human and mouse transcription factors. *Nucleic Acids Res.*, **52**, D154–D163.

9. de Tribolet-Hardy,J., Thorball,C.W., Forey,R., Planet,E., Duc,J., Coudray,A., Khubieh,B., Offner,S., Pulver,C., Fellay,J., *et al.* (2023) Genetic features and genomic targets of human KRAB-zinc finger proteins. *Genome Res.*, **33**, 1409–1423.

10. Liu,N., Hargreaves,V.V., Zhu,Q., Kurland,J.V., Hong,J., Kim,W., Sher,F., Macias-Trevino,C., Rogers,J.M., Kurita,R., *et al.* (2018) Direct promoter repression by BCL11A controls the fetal to adult hemoglobin switch. *Cell*, **173**, 430–442.e17.

11. Xie,Z., Sokolov,I., Osmala,M., Yue,X., Bower,G., Pett,J.P., Chen,Y., Wang,K., Cavga,A.D., Popov,A., *et al.* (2025) DNA-guided transcription factor interactions extend human gene regulatory code. *Nature*, **641**, 1329–1338.

12. Grand,R.S., Burger,L., Gräwe,C., Michael,A.K., Isbel,L., Hess,D., Hoerner,L., Iesmantavicius,V., Durdu,S., Pregnolato,M., *et al.* (2021) BANP opens chromatin and activates CpG-island-regulated genes. *Nature*, **596**, 133–137.

13. Tu,X., Mejía-Guerra,M.K., Valdes Franco,J.A., Tzeng,D., Chu,P.-Y., Shen,W., Wei,Y., Dai,X., Li,P., Buckler,E.S., *et al.* (2020) Reconstructing the maize leaf regulatory network using ChIP-seq data of 104 transcription factors. *Nat. Commun.*, **11**, 5089.

14. Chu,Y.-H., Lee,Y.S., Gomez-Cano,F., Gomez-Cano,L., Zhou,P., Doseff,A.I., Springer,N. and Grotewold,E. (2024) Molecular mechanisms underlying gene regulatory variation of maize metabolic traits. *Plant Cell*, **36**, 3709–3728.

15. Cahn,J., Regulski,M., Lynn,J., Ernst,E., de Santis Alves,C., Ramakrishnan,S., Chougule,K., Wei,S., Lu,Z., Xu,X., *et al.* (2024) MaizeCODE reveals bi-directionally expressed enhancers that harbor molecular signatures of maize domestication. *Nat. Commun.*, **15**, 10854.

16. Wu,H., Galli,M., Spears,C.J., Zhan,J., Liu,P., Yadegari,R., Dannenhoffer,J.M., Gallavotti,A. and Becraft,P.W. (2023) NAKED ENDOSPERM1, NAKED ENDOSPERM2, and OPAQUE2 interact to regulate gene networks in maize endosperm development. *Plant Cell*, **36**, 19–39.

17. Zhang,Y., Li,Z., Liu,J., Zhang,Y. ’e, Ye,L., Peng,Y., Wang,H., Diao,H., Ma,Y., Wang,M., *et al.* (2022) Transposable elements orchestrate subgenome-convergent and -divergent transcription in common wheat. *Nat. Commun.*, **13**, 6940.

18. Rieu,P., Beretta,V.M., Caselli,F., Thévénon,E., Lucas,J., Rizk,M., Franchini,E., Caporali,E., Paleni,C., Nanao,M.H., *et al.* (2024) The ALOG domain defines a family of plant-specific transcription factors acting during Arabidopsis flower development. *Proc. Natl. Acad. Sci. U. S. A.*, **121**, e2310464121.

19. Kerstens,M., Galinha,C., Hofhuis,H., Nodine,M., Pardal,R., Scheres,B. and Willemsen,V. (2024) PLETHORA transcription factors promote early embryo development through induction of meristematic potential. *Development*, **151**.

20. Rauluseviciute,I., Riudavets-Puig,R., Blanc-Mathieu,R., Castro-Mondragon,J.A., Ferenc,K., Kumar,V., Lemma,R.B., Lucas,J., Chèneby,J., Baranasic,D., *et al.* (2024) JASPAR 2024: 20th anniversary of the open-access database of transcription factor binding profiles. *Nucleic Acids Res.*, **52**, D174–D182.

21. Weirauch,M.T., Yang,A., Albu,M., Cote,A.G., Montenegro-Montero,A., Drewe,P., Najafabadi,H.S., Lambert,S.A., Mann,I., Cook,K., *et al.* (2014) Determination and inference of eukaryotic transcription factor sequence specificity. *Cell*, **158**, 1431–1443.

22. Sebé-Pedrós,A., Ariza-Cosano,A., Weirauch,M.T., Leininger,S., Yang,A., Torruella,G., Adamski,M., Adamska,M., Hughes,T.R., Gómez-Skarmeta,J.L., *et al.* (2013) Early evolution of the T-box transcription factor family. *Proc. Natl. Acad. Sci. U. S. A.*, **110**, 16050–16055.

23. Lambert,S.A., Yang,A.W.H., Sasse,A., Cowley,G., Albu,M., Caddick,M.X., Morris,Q.D., Weirauch,M.T. and Hughes,T.R. (2019) Similarity regression predicts evolution of transcription factor sequence specificity. *Nat. Genet.*, **51**, 981–989.

24. Najafabadi,H.S., Mnaimneh,S., Schmitges,F.W., Garton,M., Lam,K.N., Yang,A., Albu,M., Weirauch,M.T., Radovani,E., Kim,P.M., *et al.* (2015) C2H2 zinc finger proteins greatly expand the human regulatory lexicon. *Nat. Biotechnol.*, **33**, 555–562.

25. Siggers,T., Reddy,J., Barron,B. and Bulyk,M.L. (2014) Diversification of transcription factor paralogs via noncanonical modularity in C2H2 zinc finger DNA binding. *Mol. Cell*, **55**, 640–648.

26. Nakagawa,S., Gisselbrecht,S.S., Rogers,J.M., Hartl,D.L. and Bulyk,M.L. (2013) DNA-binding specificity changes in the evolution of forkhead transcription factors. *Proc. Natl. Acad. Sci. U. S. A.*, **110**, 12349–12354.

27. Matys,V., Kel-Margoulis,O.V., Fricke,E., Liebich,I., Land,S., Barre-Dirrie,A., Reuter,I., Chekmenev,D., Krull,M., Hornischer,K., *et al.* (2006) TRANSFAC and its module TRANSCompel: transcriptional gene regulation in eukaryotes. *Nucleic Acids Res.*, **34**, D108–10.

28. Gerstein,M.B., Lu,Z.J., Van Nostrand,E.L., Cheng,C., Arshinoff,B.I., Liu,T., Yip,K.Y., Robilotto,R., Rechtsteiner,A., Ikegami,K., *et al.* (2010) Integrative analysis of the Caenorhabditis elegans genome by the modENCODE project. *Science*, **330**, 1775–1787.

29. Kudron,M., Gevirtzman,L., Victorsen,A., Lear,B.C., Gao,J., Xu,J., Samanta,S., Frink,E., Tran-Pearson,A., Huynh,C., *et al.* (2024) Binding profiles for 961 Drosophila and C. elegans transcription factors reveal tissue-specific regulatory relationships. *Genome Res.*, **34**, 2319–2334.

30. Skribbe,M., Soneson,C., Stadler,M.B., Schwaiger,M., Suma Sreechakram,V.N., Iesmantavicius,V., Hess,D., Moreno,E.P.F., Braun,S., Seebacher,J., *et al.* (2025) A comprehensive Schizosaccharomyces pombe atlas of physical transcription factor interactions with proteins and chromatin. *Mol. Cell*, **85**, 1426–1444.e8.

31. Mahendrawada,L., Warfield,L., Donczew,R. and Hahn,S. (2023) Surprising connections between DNA binding and function for the near-complete set of yeast transcription factors. *bioRxivorg*, 10.1101/2023.07.25.550593.

32. Wang,X., Qiu,Z., Zhu,W., Wang,N., Bai,M., Kuang,H., Cai,C., Zhong,X., Kong,F., Lü,P., *et al.* (2023) The NAC transcription factors SNAP1/2/3/4 are central regulators mediating high nitrogen responses in mature nodules of soybean. *Nat. Commun.*, **14**, 4711.

33. Jiao,W., Wang,M., Guan,Y., Guo,W., Zhang,C., Wei,Y., Zhao,Z., Ma,H., Wang,L., Jiang,X., *et al.* (2024) Transcriptional regulatory network reveals key transcription factors for regulating agronomic traits in soybean. *Genome Biol.*, **25**, 313.

34. Li,F., Wang,J., Wang,P. and Li,L. (2025) Dephosphorylation of bZIP59 by PP2A ensures appropriate shade avoidance response in Arabidopsis. *Dev. Cell*, **60**, 551–566.e6.

35. Thoris,K., Correa Marrero,M., Fiers,M., Lai,X., Zahn,I.E., Jiang,X., Mekken,M., Busscher,S., Jansma,S.,

Nanao,M., *et al.* (2024) Uncoupling FRUITFULL's functions through modification of a protein motif identified by co-ortholog analysis. *Nucleic Acids Res.*, **52**, 13290–13304.

36. Choudhary,A., Ammari,M., Yoon,H.S. and Zander,M. (2024) High-throughput capture of transcription factor-driven epigenome dynamics using PHILO ChIP-seq. *Nucleic Acids Res.*, **52**, e105.

37. Yin,L., Zander,M., Huang,S.-S.C., Xie,M., Song,L., Saldierna Guzmán,J.P., Hann,E., Shanbhag,B.K., Ng,S., Jain,S., *et al.* (2023) Transcription factor dynamics in cross-regulation of plant hormone signaling pathways. *bioRxivorg*, 10.1101/2023.03.07.531630.

38. de Los Reyes,P., Serrano-Bueno,G., Romero-Campero,F.J., Gao,H., Romero,J.M. and Valverde,F. (2024) CONSTANS alters the circadian clock in Arabidopsis thaliana. *Mol. Plant*, **17**, 1204–1220.

39. Miller,C.N., Jarrell-Hurtado,S., Haag,M.V., Sara Ye,Y., Simenc,M., Alvarez-Maldonado,P., Behnami,S., Zhang,L., Swift,J., Papikian,A., *et al.* (2025) A single-nuclei transcriptome census of the Arabidopsis maturing root identifies that MYB67 controls phellem cell maturation. *Dev. Cell*, **60**, 1377–1391.e7.

40. Castro-Mondragon,J.A., Riudavets-Puig,R., Rauluseviciute,I., Lemma,R.B., Turchi,L., Blanc-Mathieu,R., Lucas,J., Boddie,P., Khan,A., Manosalva Pérez,N., *et al.* (2022) JASPAR 2022: the 9th release of the open-access database of transcription factor binding profiles. *Nucleic Acids Res.*, **50**, D165–D173.

41. Kulakovskiy,I.V., Makeev,V.J., Grosse,I., Grau,J., Bucher,P., Kolpakov,F., Deplancke,B., Hughes,T., Vorontsov,I. and Codebook/GRECO-BIT Consortium (2023) Codebook Motif Explorer Supplementary Dataset. 10.5281/ZENODO.10182957.

42. Barrett,T., Wilhite,S.E., Ledoux,P., Evangelista,C., Kim,I.F., Tomashevsky,M., Marshall,K.A., Phillippy,K.H., Sherman,P.M., Holko,M., *et al.* (2013) NCBI GEO: archive for functional genomics data sets--update. *Nucleic Acids Res.*, **41**, D991–5.

43. Van Bel,M., Silvestri,F., Weitz,E.M., Kreft,L., Botzki,A., Coppens,F. and Vandepoele,K. (2022) PLAZA 5.0: extending the scope and power of comparative and functional genomics in plants. *Nucleic Acids Res.*, **50**, D1468–D1474.

44. Wingender,E., Schoeps,T., Haubrock,M., Krull,M. and Dönitz,J. (2018) TFClass: expanding the classification of human transcription factors to their mammalian orthologs. *Nucleic Acids Res.*, **46**, D343–D347.

45. Wingender,E., Schoeps,T., Haubrock,M. and Dönitz,J. (2015) TFClass: a classification of human transcription factors and their rodent orthologs. *Nucleic Acids Res.*, **43**, D97–102.

46. Blanc-Mathieu,R., Dumas,R., Turchi,L., Lucas,J. and Parcy,F. (2024) Plant-TFClass: a structural classification for plant transcription factors. *Trends Plant Sci.*, **29**, 40–51.

47. Jenkins,V.K., Larkin,A., Thurmond,J. and FlyBase Consortium (2022) Using FlyBase: A database of Drosophila genes and genetics. *Methods Mol. Biol.*, **2540**, 1–34.

48. Harris,T.W., Antoshechkin,I., Bieri,T., Blasiar,D., Chan,J., Chen,W.J., De La Cruz,N., Davis,P., Duesbury,M., Fang,R., *et al.* (2010) WormBase: a comprehensive resource for nematode research. *Nucleic Acids Res.*, **38**, D463–7.

49. Bradford,Y.M., Van Slyke,C.E., Ruzicka,L., Singer,A., Eagle,A., Fashena,D., Howe,D.G., Frazer,K., Martin,R., Paddock,H., *et al.* (2022) Zebrafish information network, the knowledgebase for Danio rerio research. *Genetics*, **220**.

50. Rutherford,K.M., Lera-Ramírez,M. and Wood,V. (2024) PomBase: a Global Core Biodata Resource-growth, collaboration, and sustainability. *Genetics*, **227**, iyae007.

51. Engel,S.R., Aleksander,S., Nash,R.S., Wong,E.D., Weng,S., Miyasato,S.R., Sherlock,G. and Cherry,J.M. (2025) Saccharomyces Genome Database: advances in genome annotation, expanded biochemical pathways, and other key enhancements. *Genetics*, **229**.

52. Wang,J., Al-Ouran,R., Hu,Y., Kim,S.-Y., Wan,Y.-W., Wangler,M.F., Yamamoto,S., Chao,H.-T., Comjean,A., Mohr,S.E., *et al.* (2017) MARRVEL: Integration of human and model organism genetic resources to facilitate functional annotation of the human genome. *Am. J. Hum. Genet.*, **100**, 843–853.

53. Tegenfeldt,F., Kuznetsov,D., Manni,M., Berkeley,M., Zdobnov,E.M. and Kriventseva,E.V. (2025) OrthoDB and BUSCO update: annotation of orthologs with wider sampling of genomes. *Nucleic Acids Res.*, **53**, D516–D522.

54. Castro-Mondragon,J.A., Jaeger,S., Thieffry,D., Thomas-Chollier,M. and van Helden,J. (2017) RSAT matrix-clustering: dynamic exploration and redundancy reduction of transcription factor binding motif collections. *Nucleic Acids Res.*, **45**, e119.

55. Vazquez,M., Krallinger,M., Leitner,F., Kuiper,M., Valencia,A. and Laegreid,A. (2022) ExTRI: Extraction of transcription regulation interactions from literature. *Biochim. Biophys. Acta Gene Regul. Mech.*, **1865**, 194778.

56. ChatGPT *ChatGPT*.

57. Hellwig-Bürgel,T., Rutkowski,K., Metzen,E., Fandrey,J. and Jelkmann,W. (1999) Interleukin-1beta and tumor necrosis factor-alpha stimulate DNA binding of hypoxia-inducible factor-1. *Blood*, **94**, 1561–1567.

58. Ojeda,S.R., Hill,J., Hill,D.F., Costa,M.E., Tapia,V., Cornea,A. and Ma,Y.J. (1999) The Oct-2 POU domain gene in the neuroendocrine brain: a transcriptional regulator of mammalian puberty. *Endocrinology*, **140**, 3774–3789.

59. National Center for Biotechnology Information.

60. UniProt: the universal protein knowledgebase in 2021 (2021) *Nucleic Acids Res.*, **49**, D480–D489.

61. Stelzer,G., Rosen,N., Plaschkes,I., Zimmerman,S., Twik,M., Fishilevich,S., Stein,T.I., Nudel,R., Lieder,I., Mazor,Y., *et al.* (2016) The GeneCards suite: From gene data mining to disease genome sequence analyses. *Curr. Protoc. Bioinformatics*, **54**, 1.30.1–1.30.33.

62. Avsec,Ž., Weilert,M., Shrikumar,A., Krueger,S., Alexandari,A., Dalal,K., Fropf,R., McAnany,C., Gagneur,J., Kundaje,A., *et al.* (2021) Base-resolution models of transcription-factor binding reveal soft motif syntax. *Nat. Genet.*, **53**, 354–366.

63. Shrikumar,A., Greenside,P. and Kundaje,A. (2017) Learning important features through propagating activation differences. *arXiv*.

64. Persikov,A.V., Wetzel,J.L., Rowland,E.F., Oakes,B.L., Xu,D.J., Singh,M. and Noyes,M.B. (2015) A systematic survey of the Cys2His2 zinc finger DNA-binding landscape. *Nucleic Acids Research*, **43**, 1965–1984.

65. Persikov,A.V., Rowland,E.F., Oakes,B.L., Singh,M. and Noyes,M.B. (2014) Deep sequencing of large library selections allows computational discovery of diverse sets of zinc fingers that bind common targets. *Nucleic Acids Res.*, **42**, 1497–1508.

66. Deshpande,S., Yun,C.M., Ramalingam,V., Hecht,V., Patel,A., Pampari,A., Jessa,S., Zhao,R., Wang,A. and Kundaje,A. (2025) A unified lexicon of predictive DNA sequence motifs from ENCODE transcription factor binding and chromatin accessibility assays. 10.5281/ZENODO.17123348.

67. Di Tommaso,P., Chatzou,M., Floden,E.W., Barja,P.P., Palumbo,E. and Notredame,C. (2017) Nextflow enables reproducible computational workflows. *Nat. Biotechnol.*, **35**, 316–319.

68. Ewels,P.A., Peltzer,A., Fillinger,S., Patel,H., Alneberg,J., Wilm,A., Garcia,M.U., Di Tommaso,P. and Nahnsen,S. (2020) The nf-core framework for community-curated bioinformatics pipelines. *Nat. Biotechnol.*, **38**, 276–278.

69. Bailey,T.L. and Machanick,P. (2012) Inferring direct DNA binding from ChIP-seq. *Nucleic Acids Res.*, **40**, e128.

SUPPLEMENTARY FIGURES

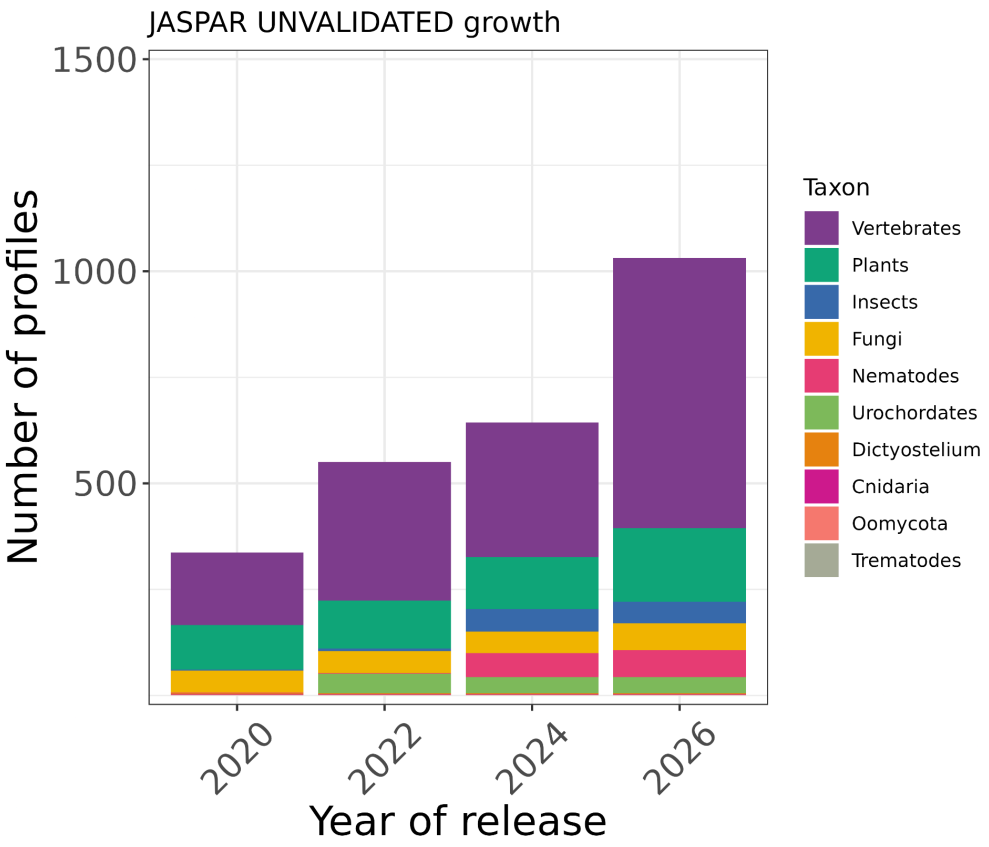

Supplementary Figure S1. Overview of the growth of the number of profiles in the JASPAR UNVALIDATED collection from the collection's introduction in 2020.

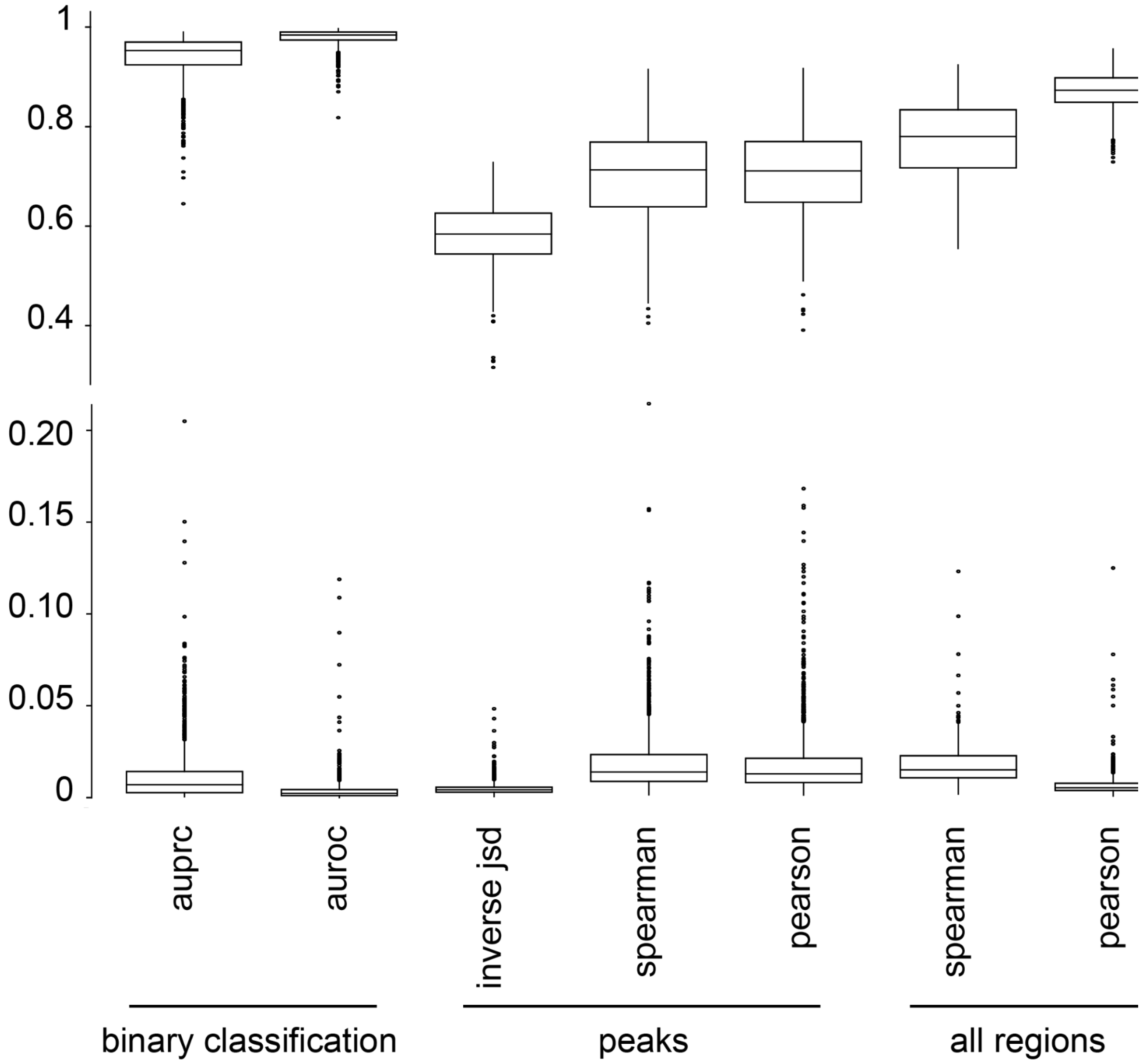

Supplementary Figure S2. BPNet model performance. Boxplots of the medians (top panel) and standard deviations (bottom panel) of performance metrics across five folds for the BPNet models included in JASPAR. AUPRC and AUROC metrics are calculated using the ChIP-seq peaks and GC-matched negative sequences (1:4) after removing all peaks (IDR-ranked peaks) from the GC-negatives. Metrics for each fold are computed on the regions in the corresponding test chromosomes for each of the folds. The inverse Jensen-Shannon Distance (JSD) is calculated by normalizing the JSD of the predicted and observed profiles between 0 and 1 (JSD with self); the higher the better for the inverse JSD. The Spearman and Pearson coefficients are calculated on the total counts, either in the peak regions or in the combined peaks and GC-negatives set. Box plots display the median as the central line, the first and third quartiles as the box, and the upper and lower whiskers extend from the quartile box to the largest/smallest value within 1.5 times the interquartile range. Outliers outside of 1.5 times the interquartile range are shown as dots.

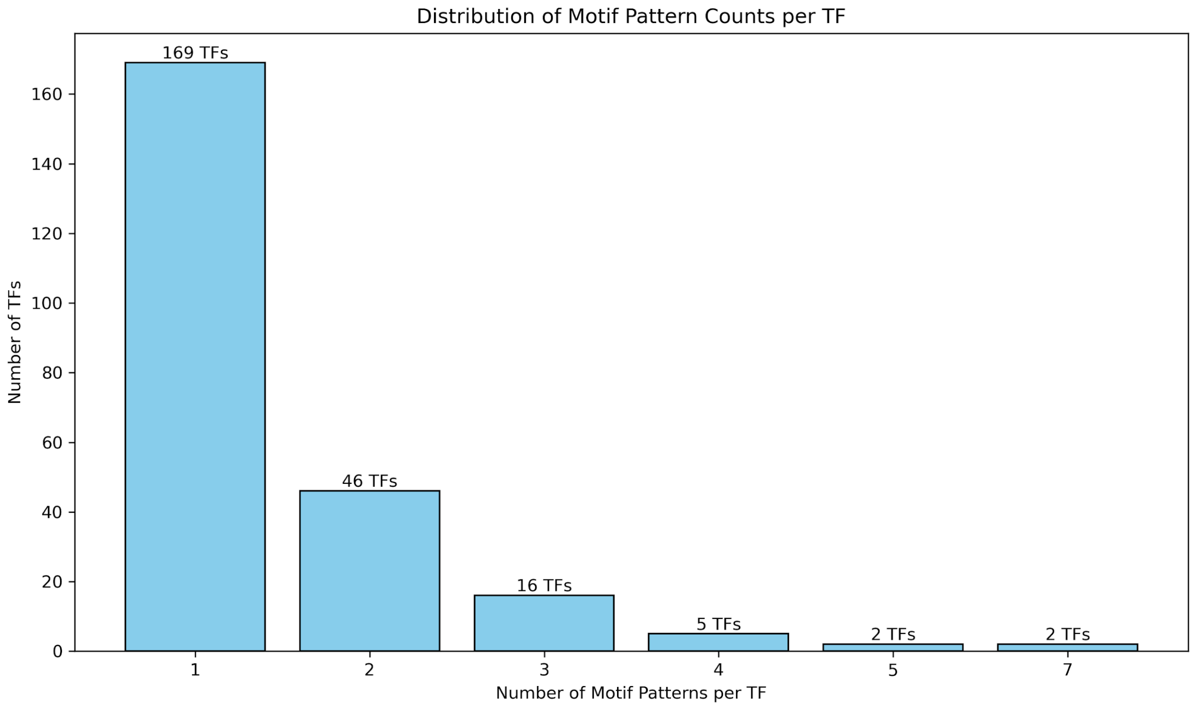

Supplementary Figure S3. Distribution of motif pattern counts per transcription factor (TF). The bar plot shows the number of TFs (y-axis) grouped by the number of distinct motif patterns identified by the clustering approach per TF (x-axis)

SUPPLEMENTARY TABLES

Supplementary Table S1. Data processed for the 11th JASPAR release (JASPAR 2026). To generate sets of PFMs for the curation, we processed multiple datasets from publicly available databases and various publications. The table lists the source of the data, the organism from which the data is coming, and the data type (experiment).

Source	Organism	Data type	Number of motifs curated	Reference
**Nordin *et al.***	*Mus musculus*	CUT&RUN	87	PMID: 40013513
**Weigel and Tegethoff *et al.***	*Mus musculus*	CUT&RUN	8	PMID: 36782060
**Fixsen *et al.***	*Mus musculus*	ChIP-seq	3	PMID: 37322178
**Codebook**	*Homo sapiens*	ChIP-seq	749	PMID: 39605320
		PBM	171	PMID: 39605729
		SMiLE-seq	150	PMID: 39605320
		HT-SELEX	579	PMID: 39605530
		GHT-SELEX	776	PMID: 39605368
**HOCOMOCO v12**	*Homo sapiens*	Collection of data types	609	PMID: 37971293
**KRABopedia**	*Homo sapiens*	ChIP-seq	147	PMID: 37730438
**Liu *et al.***	*Homo sapiens*	CUT&RUN	15	PMID: 29606353
**Xie *et al.***	*Homo sapiens*	CAP-SELEX	1,089	PMID: 40205063
**Grand and Burger *et al.***	*Homo sapiens* and *Mus musculus*	ChIP-seq	30	PMID: 34234345
**Tu and Mejía-Guerra *et al.***	*Zea mays*	ChIP-seq	275	PMID: 33037196

**Chu *et al.***	*Zea mays*	DAP-seq and ampDAP-seq	36	PMID: 38922302
**Cahn *et al.***	*Zea mays*	ChIP-seq	11	PMID: 39738013
**Wu *et al.***	*Zea mays*	DAP-seq	15	PMID: 37795691
**Zhang, Li, Liu, Zhang, and Ye *et al.***	*Triticum aestivum*	DAP-seq	498	PMID: 36376315
**Rieu, Beretta *et al.***	*Arabidopsis thaliana*	DAP-seq	21	PMID: 38412122
**Kerstens *et al.***	*Arabidopsis thaliana*	DAP-seq	3	PMID: 38884589
**Miller *et al.***	*Arabidopsis thaliana*	DAP-seq	3	PMID: 39793584
**de Los Reyes *et al.***	*Arabidopsis thaliana*	ChIP-seq	8	PMID: 38894538
**Yin *et al.***	*Arabidopsis thaliana*	ChIP-seq	45	PMID: 36945593
**Choudhary *et al.***	*Arabidopsis thaliana*	ChIP-seq	3	PMID: 39588772
**Thoris *et al.***	*Arabidopsis thaliana*	DAP-seq	15	PMID: 39475190
**Li *et al.***	*Arabidopsis thaliana*	ChIP-seq	12	PMID: 39536759
**Jiao, Wang, Guan, Guo *et al.***	*Glycine max*	DAP-seq	437	PMID: 39695844
**Wang, Qiu, Zhu, and Wang *et al.***	*Glycine max*	ChIP-seq	24	PMID: 37543605
**Pelletier *et al.***	*Glycine max*	ChIP-seq	36	GEO ID: GSE253104, GSE242528
**Lang *et al.***	*Solanum lycopersicum*	DAP-seq	681	GEO IDs: GSE172260, GSE172249
**Lang *et al.***	*Solanum lycopersicum*	ChIP-seq	12	GEO ID: GSE173208
**Mahendrawada *et al.***	*Saccharomyces cerevisiae*	ChEC-seq	534	PMID: 37546716

**Skribbe *et al.***	*Schizosaccharomyces pombe*	ChIP-seq	182	PMID: 40015273
**Kudron *et al.***	*Drosophila melanogaster*	ChIP-seq	1,784	PMID: 39438113
	Caenorhabditis elegans		1,743	
**Gerstein *et al.***	*Caenorhabditis elegans*	ChIP-seq	15	PMID: 21177976
**CIS-BP**	*Vertebrates*	Collection of data types	55	PMIDs: 25215497, 31133749
	Fungi		49	PMIDs: 16381825, 23836653, 25042805, 25215497, 25690854, 31133749
	Insects		15	PMIDs: 24043797, 25215497
	Nematodes		2	PMID: 25215497
**JASPAR 2024 UNVALIDATED collection**	Vertebrates	Collection of data types	317	PMID: 37962376
	Plants		122	
	Nematodes		57	
	Insects		53	
	Fungi		51	
	Urochordates		38	
**TOTAL**			**11,565**	

Supplementary Table S2. Overview of the JASPAR 2026 UNVALIDATED collection update.

Taxonomic group in UNVALIDATED collection	Non-redundant PFMs in JASPAR 2022	New non-redundant PFMs in JASPAR 2024	Removed PFMs	Upgraded PFMs (from UNVALI-DATED to CORE)	Downgraded PFMs (from CORE to UNVALIDATED)	Updated PFMs in JASPAR 2024	Total non-redundant PFMs in JASPAR 2024
Plants	122	56	1	16	12	-	173
Vertebrates	317	342	3	19	-	-	637
Urochordata	38	-	-	-	-	-	38
Insects	53	4	-	6	-	-	51
Nematodes	57	7	-	-	-	-	64
Fungi	51	12	-	-	-	-	63
Dictyostelium	2	-	-	-	-	-	2
Cnidaria	1	-	-	-	-	-	1
Trematodes	1	-	-	-	-	-	1
Oomycota	1	-	-	-	-	-	1
**UNVALIDATED total**	**643**	**421**	**4**	**41**	**12**	**-**	**1,031**
